# MiR-27a-5p deficiency in plasma exosomes derived from obese individuals exacerbates ventilator-induced lung injury in vitro and in vivo

**DOI:** 10.1186/s12967-025-06988-0

**Published:** 2025-11-11

**Authors:** Bailun Wang, Changping Gu, Yi Zhang, Liang Zhao, Li Yang, Yongtao Sun, Qian Chen, Daqing Ma, Yuelan Wang

**Affiliations:** 1 Department of Anesthesiology, Shandong Provincial Hospital Affiliated to Shandong First Medical University, Jinan, 250021 Shandong China; 2 Shandong University, Jinan, 250100 Shandong China; 3 Anesthesiology Department of Rizhao People’s Hospital, Rizhao, 276826 Shandong China; 4 Department of Anesthesiology and Perioperative Medicine, The First Affiliated Hospital of Shandong First Medical University, Taian, China; 5 Perioperative and Systems Medicine Laboratory, National Clinical Research Center for Child Health, Zhejiang University School of Medicine, Zhejiang, China; 6 Department of Anesthesiology, Children’s Hospital, Zhejiang University School of Medicine, Zhejiang, China; 7 Department of Anesthesiology, The First Affiliated Hospital, Ningbo University, Ningbo, China; 8 Division of Anaesthetics, Pain Medicine and Intensive Care, Department of Surgery and Cancer, Faculty of Medicine, Imperial College London, Chelsea & Westminster Hospital, London, UK

**Keywords:** VILI, Obesity, Pyk2, Exosomes, Endocytosis

## Abstract

**Background:**

Ventilator-induced lung injury (VILI) frequently occurs in obese individuals and significantly negates outcomes. Understanding mechanisms behind susceptibility of obese patients to VILI is, therefore, important. This study delved into the molecular biology mechanisms of VILI and investigated the effects of plasma exosomes from obese patients on VILI in mice, as well as its potential molecular mechanisms.

**Methods:**

MicroRNAs (miRNAs) in plasma-derived exosomes from 6 obese patients and 6 healthy volunteers were analysed to map different miRNA profiles. A lung cell line (MLE-12) was cultured with or without exosomes from obese patients and then challenged with cyclic stretching for 4 h. Western blotting, immunofluorescence, electron microscopy and other techniques were used to detect intercellular connections directly or indirectly. Co-immunoprecipitation and dual luciferase reporter experiments were conducted to verify underlying mechanism involved. Lung function, wet/dry (W/D) ratio, pathological changes, and inflammatory cytokines in alveolar lavage fluid were measured in C57BL/6 mice after 4 h of mechanical ventilation with high tidal volume.

**Results:**

Compared to those in healthy volunteers, miR-27a-5p was decreased in plasma exosomes from obese patients. Cyclic stretching induces endoplasmic reticulum (ER) stress in lung cells and consequently promotes the generation of Xbp1s, which act as transcription factors to initiate the phosphorylation of proline-rich tyrosine kinase 2 (Pyk2). p-Pyk2 thereby activated hepatocyte growth factor-regulated tyrosine kinase substrate (Hgs), which, in turn, promoted the endocytosis and transport of E-cadherin for lysosomal degradation and subsequently disrupted the pulmonary epithelial barrier. miR-27a-5p administered to cultured lung cells and mice protected lung cells and attenuated VILI through the regulation of Pyk2.

**Conclusion:**

Our findings suggest that the increased susceptibility of obese patients to VILI is likely, at least in part, due to a lack of miR-27a-5p in plasma exosomes. Targeting miR-27a-5p and its associated molecules may be a promising therapeutic avenue for preventing and treating VILI in obese patients.

**Graphical abstract:**

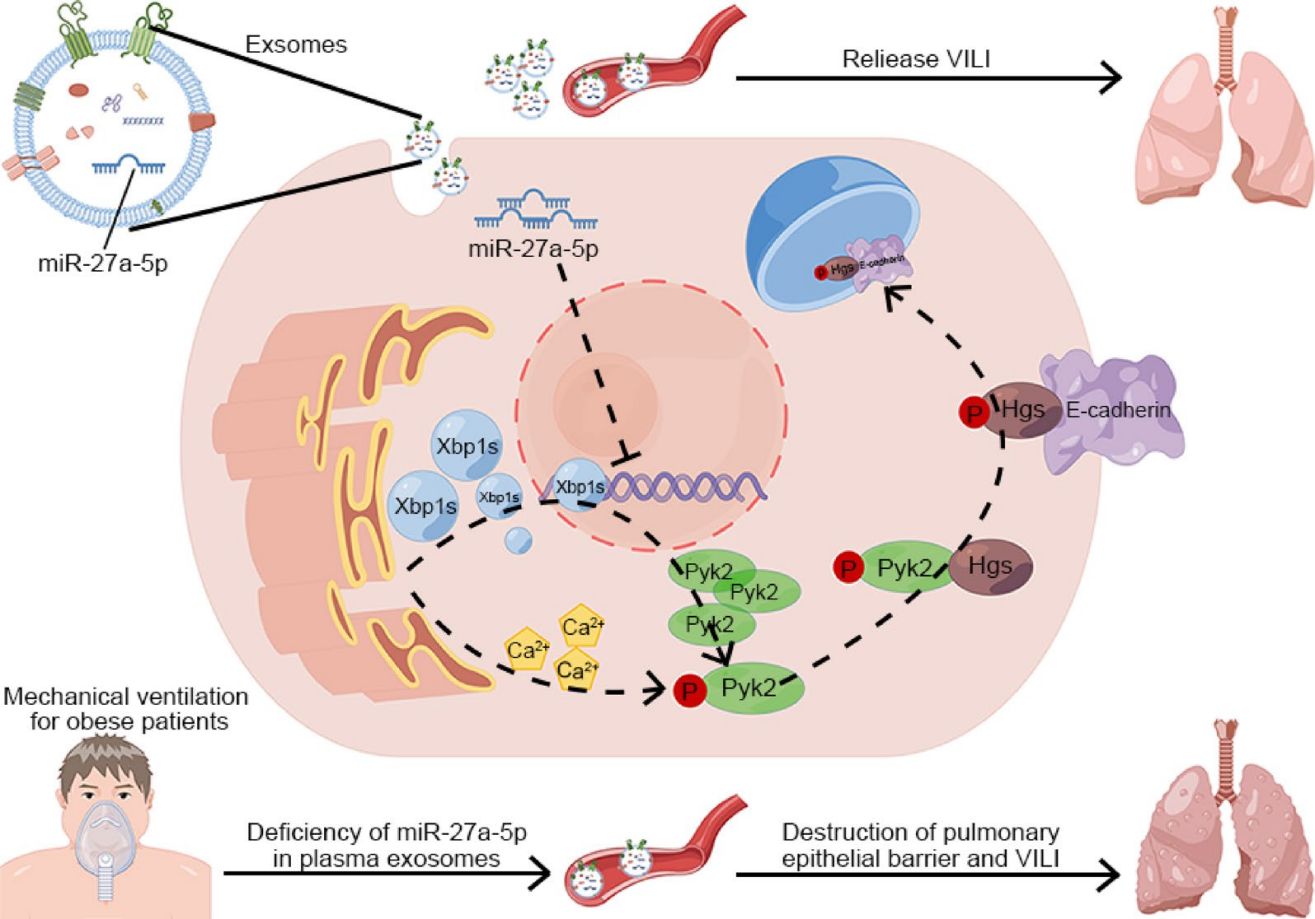

**Supplementary Information:**

The online version contains supplementary material available at 10.1186/s12967-025-06988-0.

## Highlights

Cyclic stretching breaks down the pulmonary epithelial barrier through the Xbp1/Pyk2/Hgs modulation.

miR-27a-5p reduces ventilator-induced lung injury by regulating Pyk2.

miR-27a-5p deficiency in plasma exosomes derived from obese patients makes them more susceptible to VILI.

Targeting miR-27a-5p and its associated molecules may prevent and/or treat VILI in obese patients.

**Supplementary Information:**

The online version contains supplementary material available at 10.1186/s12967-025-06988-0.

## Background

Mechanical ventilation is crucial for treating patients with acute lung injury, acute respiratory distress syndrome, and respiratory failure [1, 2]. Nonetheless, improper mechanical stress may cause airway and alveolar damage, known as VILI [3], which is a major complication in mechanically ventilated patients and is primarily characterized by increased alveolar membrane permeability [4], inflammatory cell infiltration and cytokine release [5]. The increase in alveolar membrane permeability is attributed to destruction of the alveolar membrane and a decrease in the cell membrane connexin content [6]. Periodic mechanical stretching alters the structure of alveolar epithelial cells, leading to dysfunction of the alveolar epithelial barrier [7]. A reduction in the expression of adhesion proteins, including E-cadherin, which leads to the disruption of intercellular connections, is a primary cause of epithelial barrier disorders [8].

Proline-rich tyrosine kinase 2 (Pyk2), a highly active tyrosine protein kinase, is expressed in various organs, including the nervous system, hematopoietic system, small intestines, kidney, spleen, lungs, and others [9]. When cells are subjected to extracellular stress or mechanical tension, the intracellular Ca^2+^ concentration is rapidly increased, thereby activating Pyk2 [10, 11]; Activated Pyk2 (p-Pyk2) can then phosphorylate vascular endothelial cadherin (VE-cadherin) in endothelial cells and promote the degradation of various adhesion proteins and disrupting intercellular connections [12]. Hepatocyte growth factor-regulated tyrosine kinase substrate (Hgs) is a protein coding gene. The protein encoded by this gene regulates endosomal sorting and plays a critical role in the recycling and degradation of membrane receptors. The encoded protein sorts monoubiquitinated membrane proteins into the multivesicular body, targeting these proteins for lysosome-dependent degradation. Previous studies showed that Hgs depletion was associated with the up-regulation of E-cadherin and reduced beta-catenin signaling and the aberrant accumulation of E-cadherin most likely resulted from impaired E-cadherin degradation in lysosomes [13]. Endoplasmic reticulum (ER) stress, an ubiquitous pathological mechanism [14], leads to cell death or apoptosis when this process is persistent or severe [15]. GRP78 is the main control molecule partner of the Hsp70 family residing in the endoplasmic reticulum. It can prevent immature proteins from leaving the endoplasmic reticulum prematurely, and is also involved in maintaining calcium homeostasis, recognizing ERAD targets, and regulating viral invasion. Its expression level is often used as an ER stress marker. X-box binding protein 1 (XBP1s) is extensively generated under ER stress and regulates downstream molecules [16]. Therefore, Pyk2, Hgs and Xbp1 directly or indirectly interact with each other *via* plasma exosomes and ultimately may affect the amount and function of E-cadherin and, ultimately, affect organ ultra-structure and function [17].

Obesity was reported to be a risk factor of acute lung injury and acute respiratory distress syndrome and to be associated with pulmonary fibrosis [18]. However, the impact of obesity on the lungs under mechanical ventilation conditions remains elusive and warrants further study. This study was designed to investigate the effects of plasma exosomes from obese patients on VILI in mice and the underlying molecular mechanisms, with a focus on the interaction of Pyk2, E-cadherin and miR-27a-5p both in vitro and in vivo.

## Methods

This study protocol was approved by the Ethics Committee and Animal Welfare Committee of the First Affiliated Hospital to Shandong First Medical University, Jinan, Shandong, China. The animal study part is also compliance with ARRIVE guideline. The brief experimental methods were presented below and detailed methodology can be found in the online supplement (methods part).

### Bioinformatics analysis

A cell model of VILI was established by cyclic stretching of human pulmonary microvascular endothelial cells (HPMECs), followed by transcriptome analysis using an Affymetrix Human Transcriptome Array 2.0. The microarray data has been deposited into a public domain (accession: GSE166772). A mechanically stretched sample of human non-small cell lung cancer cell line (A549) was obtained from the gene expression profile of GSE1541. Subsequently, the results from the dataset were evaluated and analyzed using R software.

### In vitro experiments

#### Cell culture and cyclic stretching

The lung cell line (MLE-12), the bronchial epithelial cell line (BEAS-2B) (Shanghai Zhong Qiao Xin Zhou Biotechnology, Shanghai, China) and Human Embryonic Kidney cell line (293T) (Servicebio company, Wuhan, China) were cultured, respectively. At 90% confluence, cultured MLE-12 or BEAS-2B cells were placed in a cell stretch stress loading system (Flexcell, North Carolina, USA) at 0.5 Hz and 20% stretch with a stretch relaxation ratio of 1:1.

#### Transfection

When the cell density reached 70%, MLE-12 cells and BEAS-2B cells were transfected with 0.02 OD/ml of si-Pyk2 or miR-27a-5p with Lipofectamine 3000. The transfection efficiency was verified by western blot analysis.

#### Immunofluorescence staining

After stretching, the BEAS-2B cells were fixed with 4% paraformaldehyde for immunofluorescent staining and then imaged with a laser confocal microscopy. All antibodies used in this and other part are listed in online supplemental Table S1.

#### Western blotting and immunoprecipitation

MLE-12 cells and lung tissue were lysed in RIPA buffer supplemented with 1 mM protease inhibitor, 1 mM sodium orthovanadate, and 1 mM PMSF. After ultrasonication, protein concentration in the supernatant was measured using a BCA protein concentration determination kit for further Western blotting and immunoprecipitation analysis.

#### RNA isolation and qRT‒PCR

RNA was extracted with a Fastagen RNA Extraction Kit (Shanghai, China) from treated MLE-12 cells. RT‒qPCR analysis was conducted on a Light Cycler instrument (Bio-Rad, California, USA) with the FastStart Essential DNA Green Master Kit (Roche, Basel, Switzerland). The expression of the target genes was normalized with the housekeeping gene GAPDH using the 2 − ΔΔCT method. The sequences of the primers used are listed in online supplemental Table S2.

#### Dual luciferase reporter assay

293T cells were seeded in triplicate into a 96-well plate at 70% confluence and transfected with luciferase reporter and transcription factor plasmids using Lipofectamine 2000 after 24 h culture in accordance with the manufacturer’s instructions. Firefly, luciferase and Renilla luciferase signals were detected at 48 h after transfection using a dual luciferase reporting kit. Relative promoter activity is expressed as the ratio of firefly to Renilla luciferase activity. The sequences of the Pyk2 promoter and mutants driving luciferase expression are listed in online supplemental Table S3.

#### Transmission electron microscopy

After fixation, rinsing, dehydration, infiltration, embedding and sectioning, the processed MLE-12 cells were subjected to transmission electron microscopy (TEM) (HT-7800, Hitachi, Japan) assessment.

#### Molecular docking

Both the Hgs (3ZYQ) and Pyk2 (3CC6) crystal structures were reported in the Protein Data Bank database (https://www.rcsb.org/). The Docking Web Server (GRAMM) (https://gramm.compbio.ku.edu/) was used for molecular docking [19]. The Hgs and Pyk2 interaction diagram was generated through PyMOL.

#### Exosome isolation and characterization

Extracellular vesicles were extracted from blood samples collected clinically using ultracentrifugation [20]. The extracted plasma exosomes were labeled with PKH67 (Beijing Fluorescence Biotechnology Co. Ltd, Beijing, China), added to MLE-12 cells for 12 h, and observed *via* laser confocal microscopy. MLE-12 cells were cultured in the presence of extracellular vesicles, which were derived from both normal controls and obese patients at a concentration of 20 µg/mL, for a period of 24 h [21].

### In vivo experiments

#### Animals

C57BL/6 mice (Male, 5–6 weeks old) were obtained from Beijing Vital River Laboratory Animal Technology (Beijing, China).

#### Liposome preparation and in vivo gene delivery

Two days after liposome solutions *via* the tail vein injection, si-Pyk2 or miR-27a-5p at 1.5 OD was injected again. The sequences of the miRNAs and siRNAs used are listed in online supplemental Table S4. One hundred micrograms of plasma exosomes were injected into mice before mechanical ventilation [22].

#### Mechanical ventilation and measurements

Seventy-eight mice were randomly divided into 13 experimental groups (*n* = 6/group) and the grouping details were presented in online supplemental Table S5. After mechanical ventilation, mice were connected to the flexiVent system (SCIREQ) to detect static lung compliance (Cst), elastance (Ers), and tissue damping (G) [23]. Lung injury was then assessed by analyzing proinflammatory factors in bronchoalveolar lavage fluid (BALF), the lung W/D ratio, HE and immunohistochemical staining, and E-cadherin expression.

### Human blood samples

After obtained written informed consent form, blood samples from 6 healthy volunteers and 6 obese patients (their demographics see online supplement Table S6), who met inclusion criteria at the First Affiliated Hospital of Shandong First Medical University, were collected. The sample size was referred to a previous study where blood samples harvested from 6 human subjects for plasma exosome determination were well enough for expected statistically difference of miRNA expression [24]. Their plasma exosome miRNA sequencing (miRNA-seq) was analyzed *via* the Illumina HiSeq high-throughput sequencing platform (Genesky Biotechnologies Inc., Shanghai, China) reported in our previous study [25] and the derived dataset were further analysed in the current study.

### Statistical analyses

All results are expressed as the mean ± standard deviation (SD). One-way or two-way analysis of variance (ANOVA) followed by *post hoc* Newman-Keuls test for multi-comparison or Student’s t test was used for comparison between two groups as appropriate with GraphPad software. A p value less than 0.05 was considered to be of a statistical significance.

## Results

### Mechanical ventilation induced pulmonary edema and lung injury

**Fig. 1:**
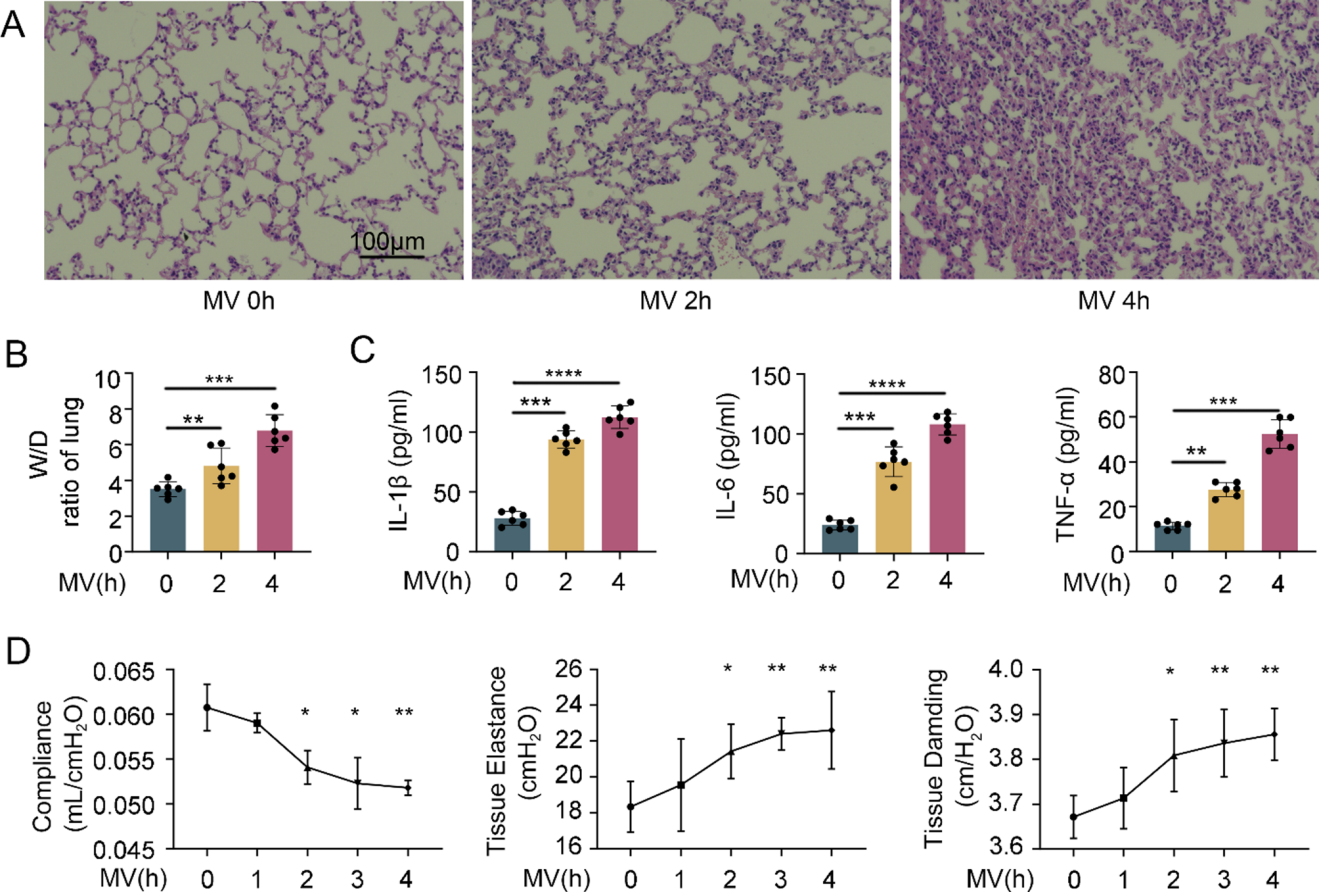
Mechanical ventilation induced lung injury in mice. (**A**) HE staining showing the histological results of lung tissue in mice after mechanical ventilation and nonmechanical ventilation. Scale bar: 100 μm. (**B**) W/D ratio of lung tissue in mechanically ventilated and nonmechanically ventilated mice. (**C**) The expression levels of IL-1β, IL-6 and TNF-α in the alveolar lavage fluid of mechanically ventilated and nonmechanically ventilated mice. (**D**) A pulmonary function detector was used to detect the compliance, tissue elasticity, and respiratory resistance of mouse lungs. The data are presented as the means (SD), *n* = 6; *p < 0.05, **p < 0.01, ***p < 0.005 and ****p < 0.001

C57BL/6 mice were subjected to mechanical ventilation for 0, 2, or 4 h. The notable changes in the post-ventilated lungs were thickened alveolar septa, dilated alveolar wall blood vessels, increased alveolar exudation, and compromised overall alveolar structure. These changes were more pronounced with longer durations of mechanical ventilation (Fig. 1A). Compared with the control group, mechanical ventilation significantly increased the lung W/D ratio of the mice from 3.5 to 6.5, i.e., increased pulmonary edema (Fig. 1B). Furthermore, proinflammatory cytokines such as IL-1β, IL-6, and TNF-α were significantly increased in the BALF (Fig. 1C). Lung compliance decreased, and lung elasticity and injury significantly increased (Fig. 1D). These findings collectively suggest that mechanical ventilation has a detrimental effect on lung function.

### Cyclic stretching reduced the expression of E-cadherin in cultured epithelial cells

**Fig. 2:**
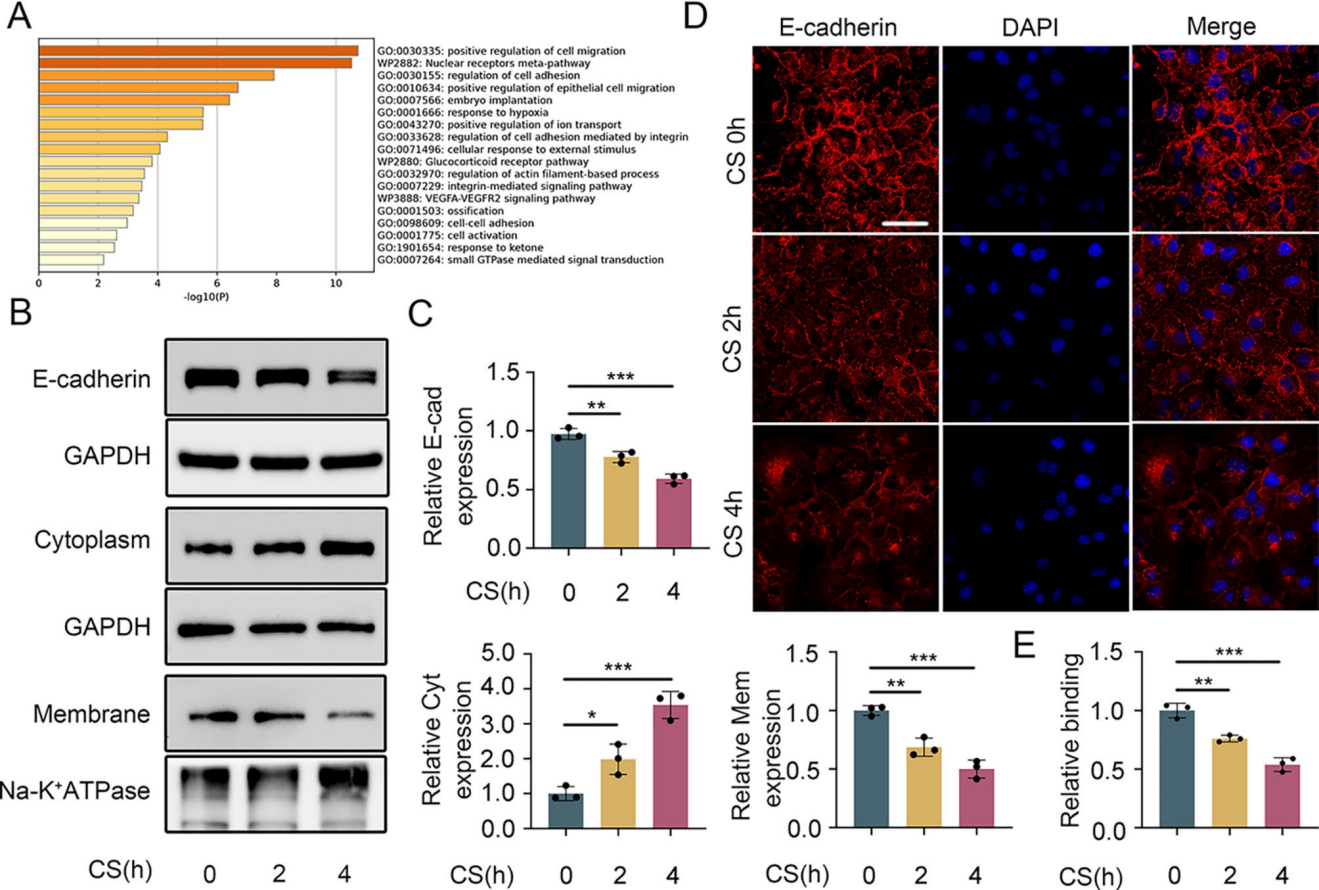
Cyclic stretching reduces the total amount and membrane expression of E-cadherin in the cytoplasm while increasing the expression of E-cadherin in the cell membrane. (**A**) GO enrichment was used to analyze changes in HPMECs before and after stretching. (**B** and **C**) Western blot results showing the total expression level of E-cadherin, its expression level in the cell membrane, and its expression level in the cytoplasm. (**D** and **E**) Immunofluorescence staining results of differently treated BEAS-2B cells. Blue: DAPI; red: E-cadherin; scale bar: 30 μm. The data are presented as the mean (SD), *n* = 3; *p < 0.05, **p < 0.01, ***p < 0.005 and ****p < 0.001

To determine the underlying mechanisms of mechanical ventilation-induced lung injury, we utilized the Affymetrix Human Transcriptome Array 2.0 for high-throughput analysis. Following data preprocessing, we identified 47 DEGs (online supplemental Table S7). To understand the biological processes and pathways influenced by these DEGs, we conducted a Gene Ontology (GO) functional enrichment analysis (Fig. 2A) Notably, cyclic stretching also altered the E-cadherin distribution in MLE-12 cells, reducing its presence in the cell membrane while increasing its cytoplasmic expression (Fig. 2B and C). The immunofluorescence data also showed a decrease in E-cadherin in the cell membrane following cyclic stretching (Fig. 2D and E). These findings demonstrate that E-cadherin expression is decreased in lung injury during mechanical ventilation.

### An increase in and activation of Pyk2 reduced E-cadherin expression, contributing to VILI

**Fig. 3:**
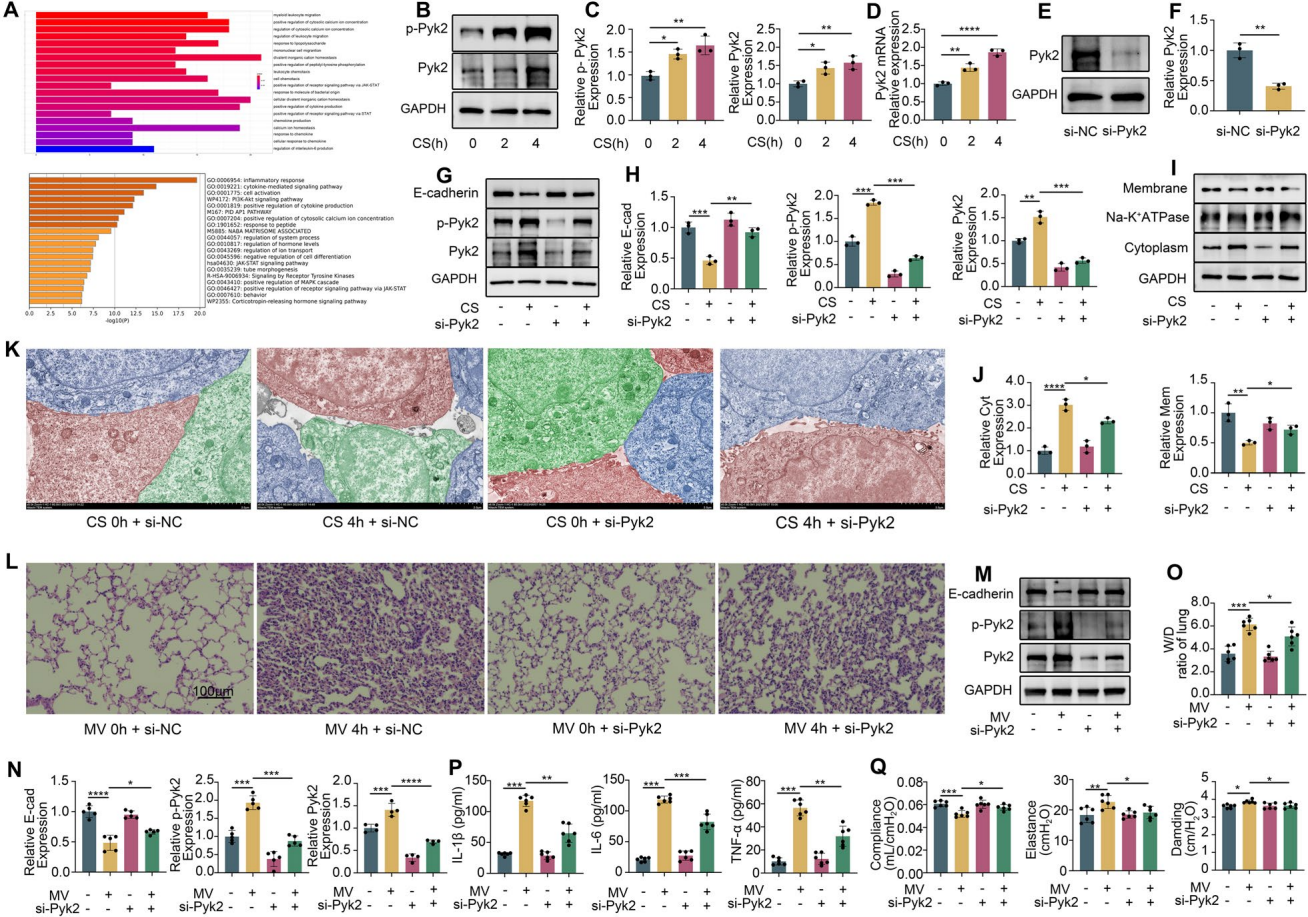
Cyclic stretching increased the expression and activation of Pyk2, which decreased E-cadherin expression. (**A**) GO enrichment analysis of the differences in A549 cells before and after stretching. (**B** and **C**) Western blotting was used to detect the expression and activation of Pyk2 after stretching. (**D**) The mRNA expression of Pyk2 was detected by qPCR after 0, 2, and 4 h of stretch. (**E** and **F**) Transfection efficiency of si-Pyk2. (**G** and **H**) Western blot results showing the expression of E-cadherin, p-Pyk2, and Pyk2 in the different groups. (**I** and **J**) Western blot results showing the expression of E-cadherin in the cell membrane and cytoplasm in the different groups. (**K**) The connections between MLE-12 cells were observed using electron microscopy. Each cell is distinguished by different colors. Scale bar: 2 μm. (**L**) HE staining showing the histological changes in the lung tissue of the mice in the different groups. Scale bar: 100 μm. (**M** and **N**) Western blot results showing the expression of E-cadherin in the lung tissues of mice in different groups. (**O**) Lung W/D ratio showing pulmonary edema in mice in the different groups. (**P**) The levels of the inflammatory factors IL-1β, IL-6 and TNF-α in the alveolar lavage fluid of mice in the different groups. (**Q**) Detection of lung compliance, tissue elasticity, and respiratory resistance in different groups of mice using lung function detectors. The data are presented as the mean (SD), *n* = 3–6; *p < 0.05, **p < 0.01, ***p < 0.005 and ****p < 0.001

We performed a differential analysis the integrated dataset (GSE27128) (online supplemental Table S8). GO functional enrichment analysis of these DEGs highlighted enrichment in receptor tyrosine kinases and calcium ion signaling pathways (Fig. 3A). Both the phosphorylation of Pyk2 and the total expression of Pyk2 increased after 4 h of cyclic stretching (CS) in MLE-12 cells (Fig. 3B and C). The mRNA expression level of Pyk2 was also increased significantly (Fig. 3D). To further investigate the relationship between Pyk2 and E-cadherin, we assessed E-cadherin expression after Pyk2 knockdown using siRNA against Pyk2. At 48 h post transfection, Pyk2 expression in the si-Pyk2 group was decreased compared with the control group (Fig. 3E and F). Notably, compared with si-control cyclic stretching (4 h), si-Pyk2 + CS (4 h) mitigated the cyclic stretching-induced decrease in E-cadherin expression (Fig. 3G and H) and increased membrane E-cadherin expression while decreasing its cytoplasmic content (Fig. 3I and J). Electron microscopy revealed that si-Pyk2 attenuated the expansion of intercellular gaps in MLE-12 cells caused by stretching (Fig. 3K).

Our in vivo experiments revealed increased Pyk2 expression and phosphorylation in mouse lung tissue in response to mechanical ventilation at 2 and 4 h (Figure S1A and S1B). The expression of Pyk2 was successfully downregulated by si-Pyk2 (Figure S1C and S1D). si-Pyk2 treatment alleviated lung pathological changes such as alveolar wall vascular dilation, congestion, alveolar destruction, and lung injury, compared to the si-control mechanical ventilation group (Fig. 3L). Moreover, E-cadherin expression, which was decreased with mechanical ventilation, was preserved to a greater extent in the si-Pyk2-treated group (Fig. 3M and N). Additionally, the si-Pyk2 group exhibited a lower lung W/D ratio and lower expression of the proinflammatory cytokines IL-1β, IL-6, and TNF-α in the BALF than the mechanical ventilation group (Fig. 3O and P). Lung function tests indicated improved compliance and significantly reduced tissue elasticity and tissue damage in the si-Pyk2 mechanical ventilation group (Fig. 3Q). These results demonstrated that inhibiting Pyk2 expression in mice effectively mitigated lung injury caused by mechanical ventilation.

### Pyk2 activated Hgs to degrade E-cadherin

**Fig. 4:**
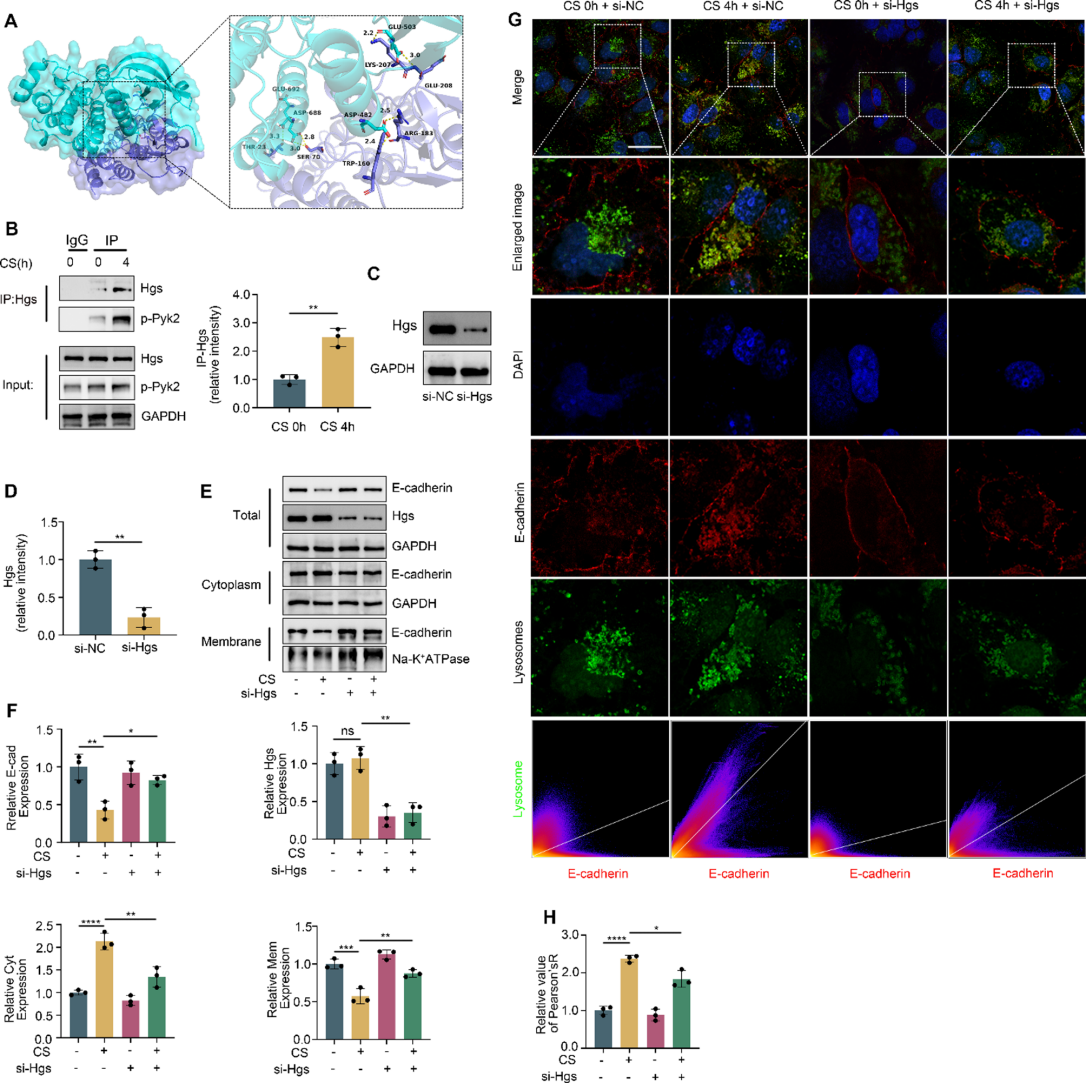
Pyk2 activated Hgs and transported E-cadherin for lysosomal degradation. (**A**) PyMol displays the molecular docking results. The Hgs protein is represented as a slate cartoon model, the Pyk2 protein is shown as a cyan cartoon model, and their binding sites are shown as the corresponding- colored stick structure. (**B**) Co-IP showed that Pyk2 bound to Hgs. (**C** and **D**) Western blot analysis verified the transfection efficiency of si-Hgs. (**E** and **F**) Western blot results showing the expression levels of E-cadherin in the entire cell, cytoplasm, and membrane of the different groups. (**G** and **H**) Colocalization of E-cadherin and lysosomes in different groups is displayed by confocal microscopy. Blue: DAPI; red: E-cadherin; green: lysosome, scale bar: 15 μm. The data are presented as the mean (SD), *n* = 3; *p < 0.05, **p < 0.01, ***p < 0.005 and ****p < 0.001

To elucidate the mechanism of Pyk2-mediated E-cadherin reduction, we employed molecular docking, coimmunoprecipitation and immunofluorescence confocal microscopy. Protein‒protein interaction analysis was then performed by using PyMOL. Notably, multiple groups of residues, such as the hydrogen bonds between THR23 of Hgs and GLU692 of Pyk2, were involved in hydrogen bonding interactions. These interactions led to a docking score of -518 for Hgs-Pyk2 (Fig. 4A). Using coimmunoprecipitation, Pyk2 was found to bind directly to Hgs and this binding increased significantly after cyclic stretching for 4 h (Fig. 4B). After 48 h si-Hgs transfection, Hgs expression was decreased compared with the control group (Fig. 4C and D). Compared with the CS (4 h) group, the si-Hgs + CS (4 h) group exhibited increased E-cadherin expression, particularly in the cell membrane, while its cytoplasmic expression decreased (Fig. 4E and F). Confocal microscopy revealed that after cyclic stretching for 4 h, the membrane content of E-cadherin almost completely decreased, and E-cadherin aggregated near lysosomes, a process mitigated by si-Hgs transfection (Fig. 4G and H). These results suggest that the binding of Pyk2 facilitates the transport of E-cadherin to lysosomes for degradation.

### The ER stress-induced Xbp1s axis increased the expression and activation of Pyk2

A significant increase in Grp78 and Xbp1s expression was noted after 4 h of cyclic stretching (Figure S2A and S2B). Furthermore, after treatment with the ER stress inhibitor 4-PBA, the expression of Grp78, Xbp1s, Pyk2, and p-Pyk2 decreased compared to that in the CS (4 h) group, and there was a concurrent increase in the total expression (Figure S2C and S2D) and membrane expression of E-cadherin. There was a reduction in cytoplasmic E-cadherin expression in the 4-PBA + CS (4 h) group (Figure S2E and S2F). This was the case for immunostaining (Figure S2G and S2H). Similarly, the expression levels of Grp78 and Xbp1s in the lung tissue of mice were increased after mechanical ventilation for 2 and 4 h (Figure S1A and S1B). This suggested that the increase in Pyk2 expression and activation and the subsequent decrease in E-cadherin induced by cyclic stretching were related to ER stress.

**Fig. 5:**
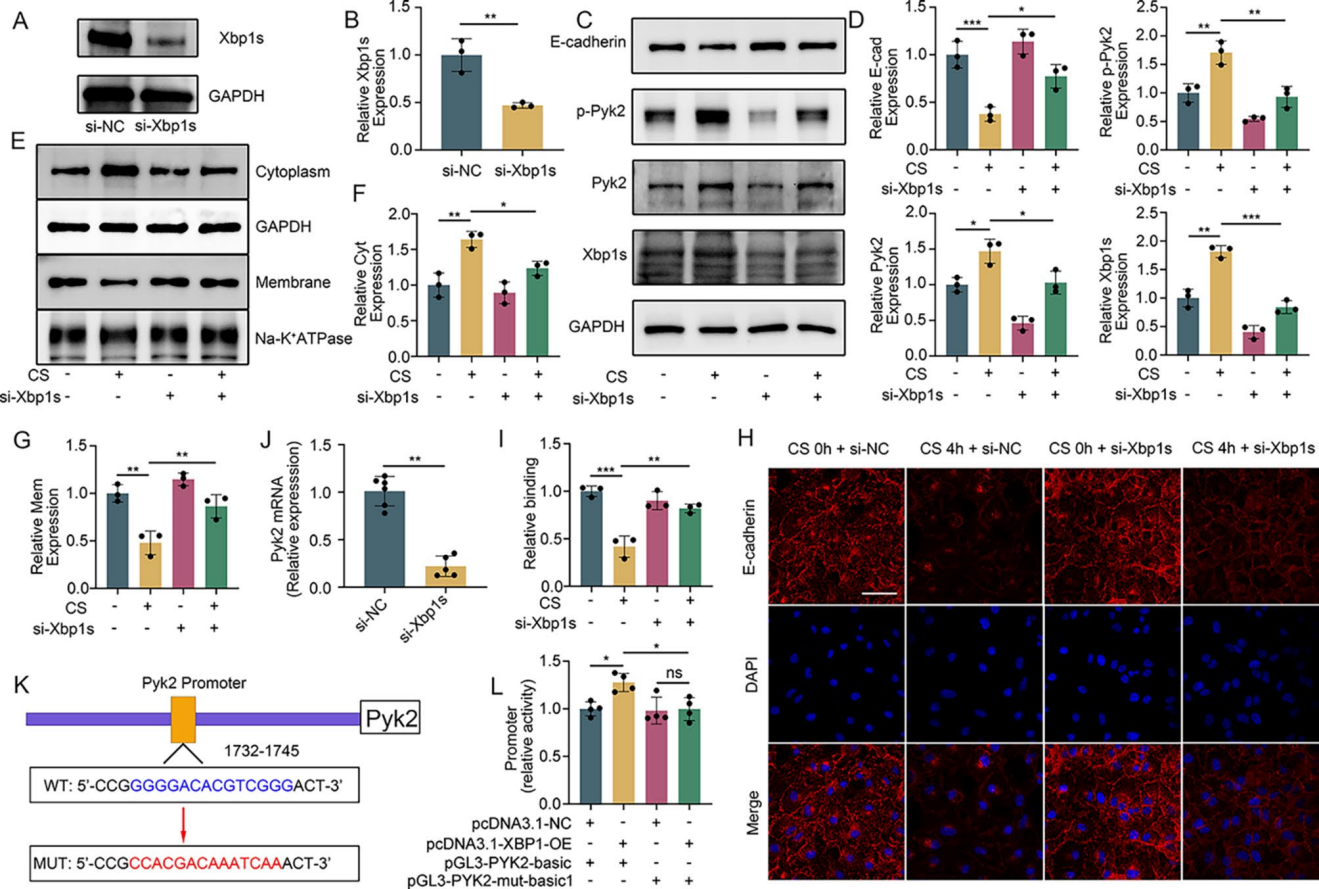
Xbp1s, a transcription factor of Pyk2, promotes Pyk2 expression and regulates E-cadherin degradation. (**A** and **B**) The transfection efficiency of si-Xbp1s was verified by western blotting. (**C** and **D**) Western blot analysis of Pyk2, p-Pyk2, and E-cadherin expression in the different groups. (**E–G**) Western blot showing the expression of E-cadherin in the cell membrane and cytoplasm in the different groups. (**H** and **I**) Immunofluorescence was used to verify the effect of Xbp1s on E-cadherin expression. Blue: DAPI. Red: E-cadherin, scale bar: 30 μm. (**J**) qPCR results showing that the mRNA expression level of Pyk2 decreased after Xbp1s was knocked down. (**K** and **L**) Dual luciferase reporter assays confirmed that Xbp1s is a transcription factor of Pyk2. The data are presented as the mean (SD), *n* = 3–6, *p < 0.05, **p < 0.01 and ***p < 0.005

Next, we depleted Xbp1s in MLE-12 cells by transfection with si-Xbp1s for 48 h. Compared with that of the control group, the expression of Xbp1s of the si-Xbp1s group decreased by 60% (Fig. 5A and B). Following mechanical stretching, the si-Xbp1s + CS (4 h) group exhibited lower Pyk2 and p-Pyk2 expression than the CS (4 h) group and an increase in E-cadherin expression (Fig. 5C and D), along with increased membrane E-cadherin and decreased cytoplasmic E-cadherin (Fig. 5E and G). Similar pattern changes were also shown by immunofluorescence staining (Fig. 5H and I).

To understand the mechanism by which Xbp1s regulates Pyk2, we utilized the JASPAR website (https://jaspar.genereg.net/*)* and hypothesized that Xbp1s may induce Pyk2 expression by acting as a transcription factor. Indeed, Pyk2 mRNA expression was significantly decreased following the transfection of si-Xbp1s (Fig. 5J). A dual luciferase reporter assay including Pyk2 WT or mutated promoter-driven luciferase and Xbp1 overexpression was then performed. A significant increase of luciferase activity was found in the presence of the WT promoter but not the mutated promoter upon Xbp1s overexpression (Fig. 5K and L). Taken together, our findings demonstrate that mechanical ventilation increases Xbp1s expression through ER stress, which in turn elevates Pyk2 expression and contributes to lung injury.

### Human plasma exosomes and their effects on MLE-12 cells

**Fig. 6:**
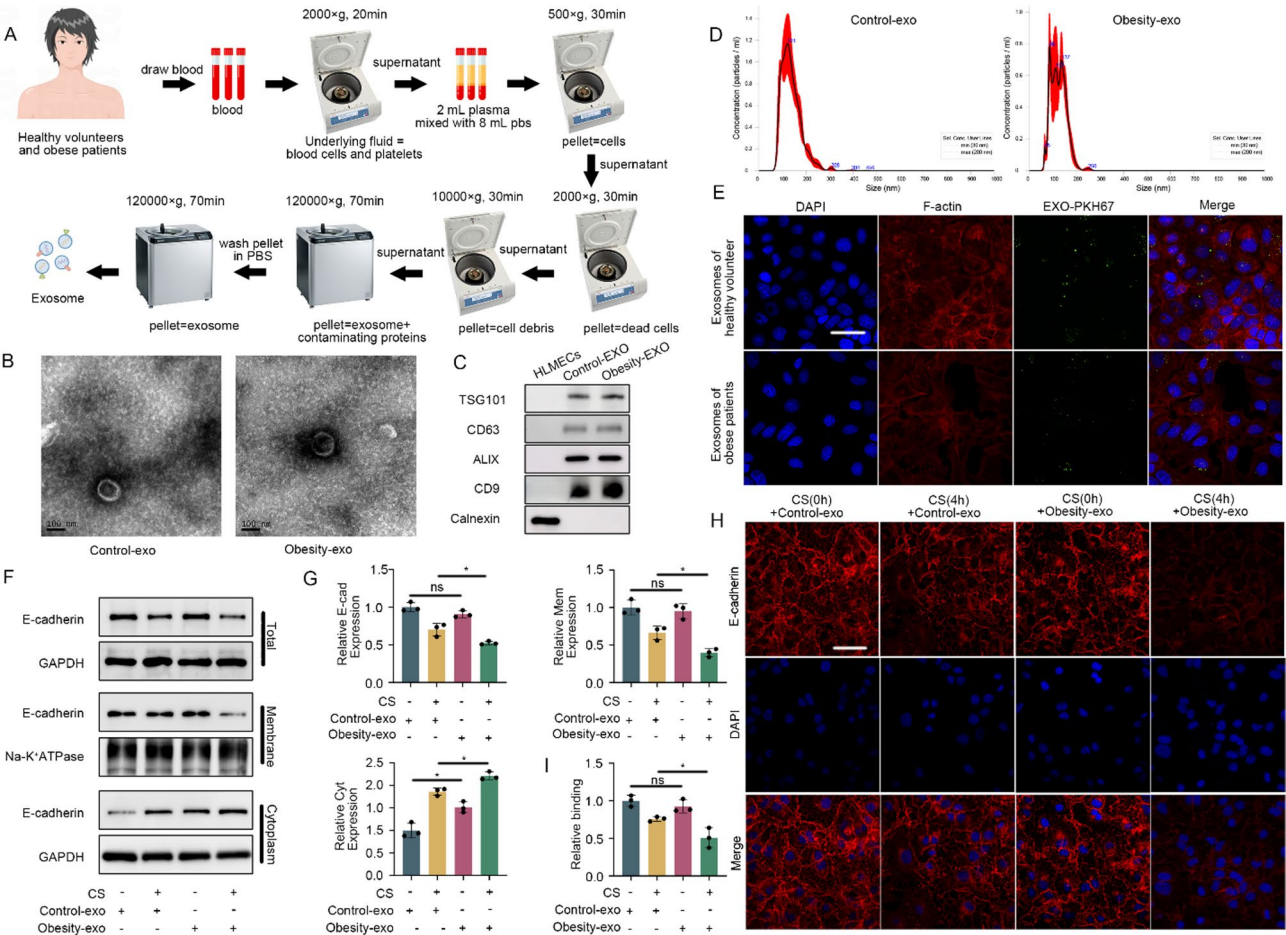
Plasma exosomes in obese patients exacerbate the reduction in E-cadherin expression induced by cyclic stretching. (**A**) Plasma exosome extraction process. (**B**) The morphology of plasma exosomes detected with transmission electron microscopy. Scale bar: 100 nm. (**C**) Detection of the TSG-101, ALIX, CD63 and CD9 proteins, as well as the expression of the invisible protein calnexin in plasma exosomes, by Western blotting. (**D**) Diameter distribution of plasma exosomes detected by nanoparticle tracking analysis. (**E**) Immunofluorescence image showing the absorption of exosomes. Blue: DAPI. Red: β-Actin. Green: exosomes; Scale bar: 30 μm. (**F** and **G**) Western blot detection of the total expression level and expression level of E-cadherin in the cell membrane and cytoplasm. (**H** and **I**) Immunofluorescence detection of E-cadherin expression and distribution in different groups. Blue: DAPI. Red: E-cadherin, scale bar: 30 μm. The data are presented as the mean (SD), *n* = 3; *p < 0.05

Plasma exosomes were isolated from obese patients and healthy volunteers (Fig. 6A). Under TEM, the vesicles exhibited a typical cup-shaped morphology with a bilayer membrane structure (Fig. 6B). The widely recognized extracellular molecular markers CD63, ALIX, CD9, and TSG101 were highly expressed in the isolated granules, while calnexin was absent (Fig. 6C). The particle diameter ranged from 50 to 150 nm (Fig. 6D). In addition, PKH67-labeled exosomes were taken up by MLE-12 cells (Fig. 6E). To investigate the impact of exosomes from obese patients on cultured lung cells, MLE-12 cells were cocultured with exosomes from obese patients and healthy volunteers and challenged with cyclic stretching. Exosomes from obese patients exacerbated the decrease in total and membrane-bound E-cadherin expression and the increase in the cytoplasmic expression of E-cadherin in MLE-12 cells induced by stretching (Fig. 6F and G). The immunofluorescence data were also in line with the western blot results (Fig. 6H and I).

### Human plasma exosomes regulated Pyk2 through miR-27a-5p

**Fig. 7:**
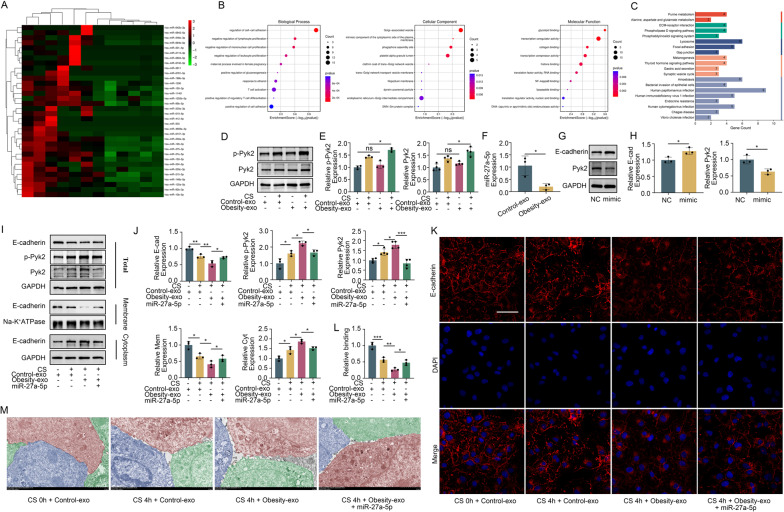
Reduced inhibitory effect of plasma exosomes on Pyk2 in obese patients due to a lack of miR-27a-5p. (**A**) Heatmap of miRNA differences in the plasma exosomes of obese patients and healthy volunteers. (**B**) GO enrichment analysis of the miR-27a-5p. (**C**) KEGG enrichment analysis of the miR-27a-5p. (**D** and **E**) Western blot analysis of the effects of plasma exosomes from obese patients and healthy volunteers on the expression and activation of Pyk2. (**F**) PCR results showing the expression of miR-27a-5p in MLE-12 cells treated with plasma exosomes from healthy volunteers and obese patients. (**G** and **H**) Western blot analysis of the effect of miR-27a-5p mimics on the expression of Pyk2 and E-cadherin. (**I** and **J**) Western blotting was used to detect the expression levels of Pyk2, p-Pyk2, total E-cadherin, and the cell membrane and cytoplasm of E-cadherin in the different groups. (**K** and **L**) Immunofluorescence detection of E-cadherin expression and distribution in MLE-12 cells in different groups. Blue: DAPI. Red: E-cadherin, scale bar: 30 μm. (**M**) Observation of the connections between MLE-12 cells in different groups via electron microscopy. Scale bar: 2 μm. The data are presented as the mean (SD), *n* = 3–4; *p < 0.05, **p < 0.01 and ***p < 0.005

Given the critical role of miRNA as a cargo in extracellular vesicles, we hypothesized that differential miRNA expression in exosomes from obese patients compared to healthy volunteers might impact lung function. We reanalyzed plasma exosome miRNA-seq dataset derived from from healthy volunteers and obese patients reported previously [25]. The 34 downregulated miRNAs were presented (Fig. 7A). By analyzing datasets from miRDB, miRTarBase, starBase and TargetScan, miR-27a-5p was found to be a putative target of Pyk2 and also it was reported that miR-27a-5p is organo-protective in sepsis induced ARDS and organ injurious models [26, 27], and therefore, it was chosen for following further experiments. Subsequently, we conducted GO and KEGG enrichment analyses of the miR-27a-5p and found that the miR-27a-5p was enriched in regulation of cell-cell adhesion, phagophore assembly site, and lysosome (Fig. 7B and C). The expression and phosphorylation of Pyk2 in MLE-12 cells treated with plasma exosomes from obese patients were greater than those in MLE-12 cells treated with plasma exosomes from healthy volunteers (Fig. 7D and E). Compared to that in MLE-12 cells treated with exosomes from healthy volunteers, the miR-27a-5p level in MLE-12 cells treated with exosomes from obese patients was significantly lower (Fig. 7F). To validate the regulatory effect of miR-27a-5p on Pyk2, we synthesized a miR-27a-5p mimic and transfected it into MLE-12 cells. The miR-27a-5p mimic significantly inhibited Pyk2 expression and enhanced E-cadherin expression (Fig. 7G and H). Furthermore, MLE-12 cells treated with plasma exosomes supplemented with a miR-27a-5p mimics decreased expression and activation of Pyk2. This treatment increased the total and membrane-bound E-cadherin while reducing its cytoplasmic expression (Fig. 7I and L). Intercellular connections in MLE-12 cells treated with exosomes from obese patients were more severely disrupted after cyclic stretching than those in MLE-12 cells treated with exosomes from healthy volunteers. This disruption was alleviated by miR-27a-5p mimics treatment (Fig. 7M). Overall, the modulation of the Pyk2/Hgs/E-cadherin pathway by plasma exosomes from obese patients is dependent on the absence of miR-27a-5p.

### Plasma exosomes from obese patients exacerbated VILI in mice due to a lack of miR-27a-5p

**Fig. 8:**
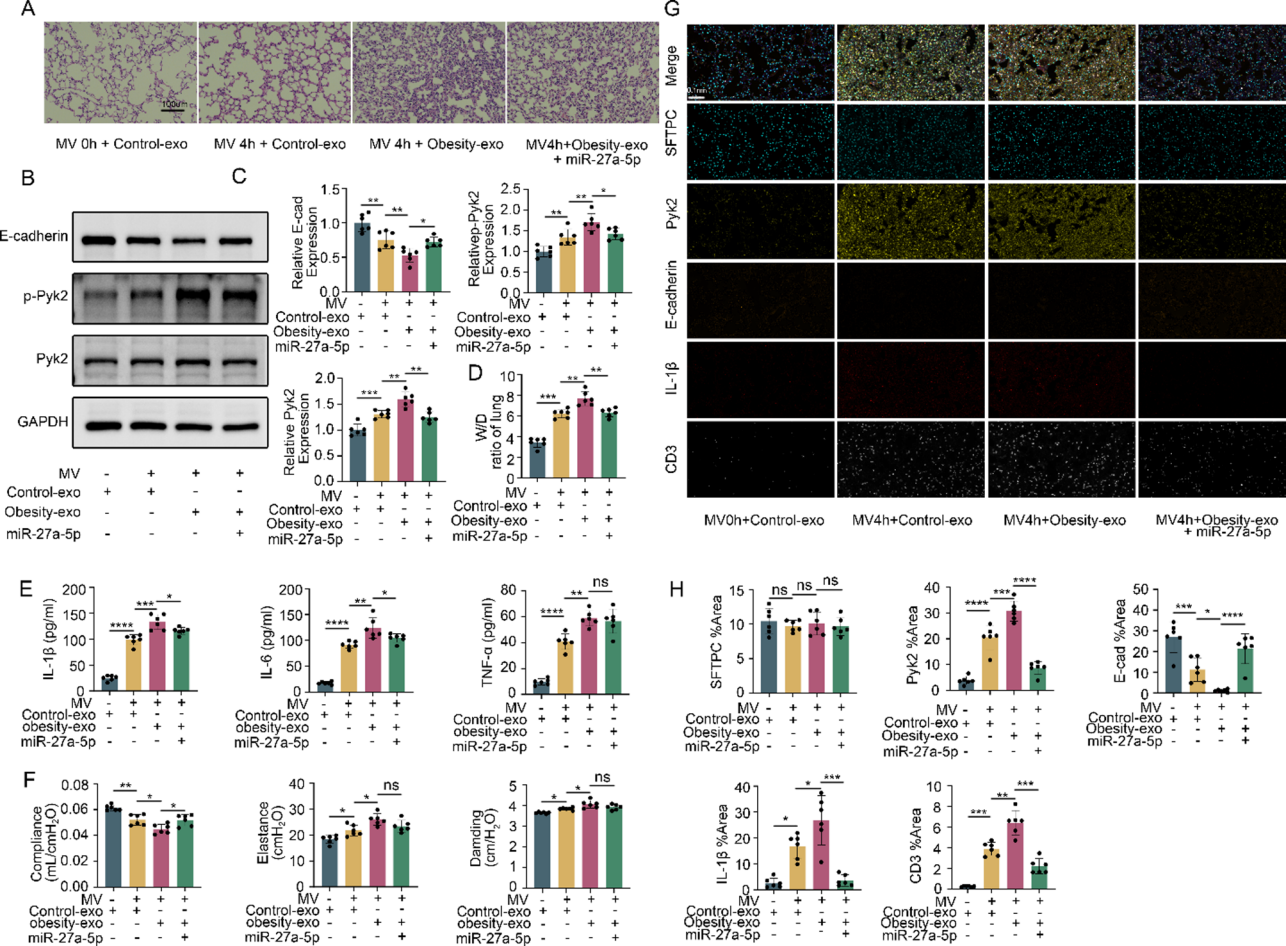
Plasma exosomes from obese patients cannot alleviate VILI in mice due to a lack of miR-27a-5p. (**A**) HE staining showing the histological results of lung tissue from mice in the different groups. Scale bar: 100 μm. (**B** and **C**) Western blot results showing the expression of E-cadherin, Pyk2, and p-Pyk2 in the lung tissues of mice in different groups. (**D**) W/D results showing pulmonary edema in mice in the different groups. (**E**) ELISA detection of IL-1β, IL-6 and TNF-α levels in the BALF of different groups of mice. (**F**) Detection of lung compliance, tissue elasticity, and respiratory resistance in different groups of mice using lung function detectors. (**G** and **H**) Multiplex immunohistochemistry was used to detect the expression of E-cadherin, Pyk2, IL-1β and CD3 in the lung tissue of mice in different groups. The data are presented as the mean (SD), *n* = 6; *p < 0.05, **p < 0.01, ***p < 0.005 and ****p < 0.001

Our cellular experiments indicated that unlike plasma exosomes from healthy volunteers, those from obese patients lack miR-27a-5p, leading to aggravated E-cadherin degradation in MLE-12 cells under stretching conditions. To further verify its role, a mechanically ventilated lung injury mouse model was established and their injured lungs were readily seen through various measurements. However, the lung injuries were more severely damaged when treated with exosomes from obese patients than that treated with exosomes from healthy volunteers. The damage was notably alleviated upon the addition of miR-27a-5p (Fig. 8A). An increase in Pyk2 expression and activation, as well as a decrease in E-cadherin expression, was detected in the lung tissue of mice treated with mechanical ventilation after receiving exosomes from obese patients. The addition of miR-27a-5p counteracted these changes (Fig. 8B and C). In addition, mechanical ventilation worsened pulmonary edema (Fig. 8D), increased inflammatory cytokines in alveolar lavage fluid (Fig. 8E), and decreased lung function in mice treated with exosomes from obese patients (Fig. 8F). These adverse effects were also mitigated by the addition of miR-27a-5p. Immunohistochemistry also further supported these findings (Fig. 8G and H).

## Discussion

In this study, we found that the lack of miR-27a-5p in plasma exosomes of obese patients promoted the internalization of E-cadherin and degradation of lysosomal pathways in alveolar epithelial cells after mechanical ventilation. All these changes ultimately led to further damage to the integrity of the lung epithelial barrier.

Mechanical ventilation is an effective supportive treatment for patients who have respiratory failure and ARDS, and widely used in intensive care settings, for example for critical COVID-19 patients [28]. However, long-term mechanical ventilation or improper ventilation may lead to VILI [29]. Previously, VILI was reported to be associated with proinflammatory release and consequent inflammatory responses [30]. However, anti-inflammatory therapy, e.g., anti-TNF-α, was not effective enough even in the preclinical setting, indicating that other mechanisms are likely involved. Our study, for the first time, showed that the increased expression and activation of Pyk2 degraded E-cadherin and subsequently disrupted the function of the pulmonary epithelial barrier, leading to VILI.

We analysed VILI-related databases and found that tyrosine kinase receptor and calcium ion signaling pathways were altered after mechanical stretching. These changes may be related to Pyk2. Therefore, we established in vivo and in vitro VILI models to investigate the role of Pyk2 and its downstream effects. We found that mechanical ventilation caused the expression and activation of Pyk2, which decreased E-cadherin expression. These findings were further supported by data generated from a series of our in vitro studies.

Hgs are important components of the endosomal sorting complex required for transport, which is involved in the intracellular sorting and transport of ubiquitination receptors [31]. Hgs were reported to be activated by tyrosine kinases to regulate E-cadherin degradation in cancer cells [13]. Our results demonstrated that p-Pyk2 bound to and activated Hgs and then mediated lysosomal E-cadherin degradation (Fig. 4), which may be one of the mechanisms by which Pyk2 mediates VILI per se.

Xbp1, involved in the ER stress response, is activated *via* an unconventional splicing mechanism [32]. Moreover, spliced Xbp1s have high stability and strong transcriptional activity [33]. Silencing Xbp1s suppresses cancer cell migration and epithelial–mesenchymal transition, whereas overexpressing Xbp1s decreases E-cadherin expression [34]. Our work revealed that Xbp1s promoted Pyk2 expression, resulting in a reduction in E-cadherin expression (Fig. 5).

Plasma exosomes in sepsis patients are involved in the occurrence and development of ALI by regulating the endothelial inflammatory response, oxidative stress, and cell apoptosis, leading to severe pulmonary vascular leakage and interstitial edema [35]. Our previous study found that the deficiency of miR-105-5p in the plasma exosomes of obese patients contributed to the disruption of the lung vascular endothelial barrier induced by mechanical ventilation *via* modulating the XBP1s/RAB7 signaling pathway [25]. In the present study, we demonstrated that the plasma exosomes of obese patients lack miR-27a-5p and, therefore, cannot inhibit the expression of Pyk2, thereby exacerbating alveolar epithelial barrier damage caused by ventilation leading to VILI. Interestingly, it has been reported that miR-27a-5p plays a crucial role in mesenchymal stromal/stem cells modulating the response to sepsis-induced lung injury *via* regulation of miR-27a-5p in recipient mice through enhancing repairing and preventing immune-cell infiltration mechanism [26]. In another report, knocking down miR-27a-5p was also found to increase the activation of NF-κB and cytokine TNF-α and IL-8, and miR-27a-5p mimics alleviated disease severity and intestinal inflammation of clostridioides difficile-infected mice [27]. These previous publications and our current work reported here with different disease models all indicate that miR-27a-5p is protective and, hence, the potential based on miR-27a-5p derived therapeutics for lung or other organ injury may be promising.

It is worth noting that mice treated with exosomes from obese patients showed increased infiltration of CD3^+^ T cells in VILI (Fig. 8G-H), which may be related to enhanced antigen exposure after epithelial barrier disruption. The supplementation of miR-27a-5p significantly reduced T cell recruitment, suggesting that it may indirectly inhibit adaptive immune activation by maintaining barrier integrity. This finding may provide a new perspective for the immune regulation of obesity related VILI.

There are some limitations in our study. For example, only male mice were used for this study and whether the pathological and molecular changes found in this study is also seen in female is unknown and, therefore, warrants further study. In addition, this study indicated that Pyk2 regulates the degradation of the adhesion protein E-cadherin, leading to destruction of pulmonary epithelial barrier function. However, whether other adhesion proteins and connexins, such as ZO-1 and occludin, also play a role remains unknown. In addition, why miR-27a-5p is absent in obese patients is unknown and further study is needed. Lastly, previous studies reported that obesity alleviated ventilator-induced lung injury through the STAT3-SOCS3 pathway [36] or that obesity even protected the lungs [37]. This discrepancy is unknown. The different experimental settings (high vs. low mechanical ventilation stretch) may be the reason; for example, fat in the chest cavity can negate intensive mechanical ventilation stretch resulting in lung injury as reported recently [38].

## Conclusion

In summary, we found that the plasma exosomes of obese patients exacerbated VILI due to the lack of miR-27a-5p in obese patients. Mechanical ventilation induces ER stress, which released a large amount of Xbp1s, a transcription factor of Pyk2, and increases Pyk2 phosphorylation due to miR-27a-5p deficiency, which then activates downstream Hgs to facilitate lysosomal degradation of E-cadherin and causes destruction of alveolar epithelial barrier function, leading to VILI development. The targeting of these molecular mechanisms reported here may shed light on the development of therapeutic strategies for preventing and treating ventilation-induced lung injury.

## Supplementary Information

Below is the link to the electronic supplementary material.

Supplementary Material 1

## Data Availability

All data generated or analysed during this study are included in this published article [and its supplementary information files].

## Abbreviations

VILI: Ventilator-induced lung injury
Pyk2: Proline-rich tyrosine kinase 2
Hgs: Hepatocyte growth factor-regulated tyrosine kinase substrate
miRNA: MicroRNAs
W/D: Wet/dry
ER: endoplasmic reticulum
ANOVA: One-way analysis of variance
SD: Standard deviation
BALF: bronchoalveolar lavage fluid
DEGs: Differentially expressed genes
HPMECs: Human pulmonary microvascular endothelial cells
GO: Gene Ontology
CS: Cyclic stretching
TEM: Transmission electron microscopy

## References

[1] Luan L, Hernandez A, Sherwood ER. Lung ventilation strategies and regional lung inflammation. Crit Care. 2013;17:184. 24007679 10.1186/cc12881 PMC4057236

[2] Pelosi P, Ball L, Barbas CSV, Bellomo R, Burns KEA, Einav S, Gattinoni L, Laffey JG, Marini JJ, Myatra SN, et al. Personalized mechanical ventilation in acute respiratory distress syndrome. Crit Care. 2021;25:250. 34271958 10.1186/s13054-021-03686-3 PMC8284184

[3] Marini JJ, Rocco PRM, Gattinoni L. Static and dynamic contributors to Ventilator-induced lung injury in clinical practice. Pressure, energy, and power. Am J Respir Crit Care Med. 2020;201:767–74. 31665612 10.1164/rccm.201908-1545CI PMC7124710

[4] Albert RK. Constant Vt ventilation and surfactant dysfunction: an overlooked cause of Ventilator-induced lung injury. Am J Respir Crit Care Med. 2022;205:152–60. 34699343 10.1164/rccm.202107-1690CP

[5] Dolinay T, Himes BE, Shumyatcher M, Lawrence GG, Margulies SS. Integrated stress response mediates epithelial injury in mechanical ventilation. Am J Respir Cell Mol Biol. 2017;57:193–203. 28363030 10.1165/rcmb.2016-0404OC PMC5576586

[6] Young BM, Shankar K, Tho CK, Pellegrino AR, Heise RL. Laminin-driven Epac/Rap1 regulation of epithelial barriers on decellularized matrix. Acta Biomater. 2019;100:223–34. 31593773 10.1016/j.actbio.2019.10.009 PMC6892605

[7] Tao Z, Jie Y, Mingru Z, Changping G, Fan Y, Haifeng W, Yuelan W. The Elk1/MMP-9 axis regulates E-cadherin and occludin in ventilator-induced lung injury. Respir Res. 2021;22:233. 34425812 10.1186/s12931-021-01829-2 PMC8382112

[8] Katsamba P, Carroll K, Ahlsen G, Bahna F, Vendome J, Posy S, Rajebhosale M, Price S, Jessell TM, Ben-Shaul A, et al. Linking molecular affinity and cellular specificity in cadherin-mediated adhesion. Proc Natl Acad Sci U S A. 2009;106:11594–9. 19553217 10.1073/pnas.0905349106 PMC2710653

[9] Ou Z, Dolmatova E, Mandavilli R, Qu H, Gafford G, White T, Valdivia A, Lassegue B, Hernandes MS, Griendling KK. Myeloid Poldip2 contributes to the development of pulmonary inflammation by regulating neutrophil adhesion in a murine model of acute respiratory distress syndrome. J Am Heart Assoc. 2022;11:e025181. 35535614 10.1161/JAHA.121.025181 PMC9238549

[10] Zhu XD, Bao YH, Guo YC, Yang WC. Proline-Rich Protein Tyrosine Kinase 2 in Inflammation and Cancer. Cancers. 2018, 10. 10.3390/cancers10050139 PMC5977112 29738483

[11] Yu H, Li X, Marchetto GS, Dy R, Hunter D, Calvo B, Dawson TL, Wilm M, Anderegg RJ, Graves LM, Earp HS. Activation of a novel calcium-dependent protein-tyrosine kinase. Correlation with c-Jun N-terminal kinase but not mitogen-activated protein kinase activation. J Biol Chem. 1996;271:29993–8. 8939945 10.1074/jbc.271.47.29993

[12] Allingham MJ, van Buul JD, Burridge K. ICAM-1-mediated, Src- and Pyk2-dependent vascular endothelial Cadherin tyrosine phosphorylation is required for leukocyte transendothelial migration. J Immunol. 2007;179:4053–64. 17785844 10.4049/jimmunol.179.6.4053

[13] Toyoshima M, Tanaka N, Aoki J, Tanaka Y, Murata K, Kyuuma M, Kobayashi H, Ishii N, Yaegashi N, Sugamura K. Inhibition of tumor growth and metastasis by depletion of vesicular sorting protein hrs: its regulatory role on E-cadherin and beta-catenin. Cancer Res. 2007;67:5162–71. 17545595 10.1158/0008-5472.CAN-06-2756

[14] Vestuto V, Di Sarno V, Musella S, Di Dona G, Moltedo O, Gomez-Monterrey IM, Bertamino A, Ostacolo C, Campiglia P, Ciaglia T. New frontiers on ER stress modulation: are TRP channels the leading actors? Int J Mol Sci. 2022;24:185. 36613628 10.3390/ijms24010185 PMC9820239

[15] Bhardwaj M, Leli NM, Koumenis C, Amaravadi RK. Regulation of autophagy by canonical and non-canonical ER stress responses. Semin Cancer Biol. 2020;66:116–28. 31838023 10.1016/j.semcancer.2019.11.007 PMC7325862

[16] Wiseman RL, Mesgarzadeh JS, Hendershot LM. Reshaping Endoplasmic reticulum quality control through the unfolded protein response. Mol Cell. 2022;82:1477–91. 35452616 10.1016/j.molcel.2022.03.025 PMC9038009

[17] Chung IM, Rajakumar G, Venkidasamy B, Subramanian U, Thiruvengadam M. Exosomes: current use and future applications. Clin Chim Acta. 2020;500:226–32. 31678573 10.1016/j.cca.2019.10.022

[18] Guo X, Sunil C, Qian G. Obesity and the development of lung fibrosis. Front Pharmacol. 2021;12:812166. 35082682 10.3389/fphar.2021.812166 PMC8784552

[19] Katchalski-Katzir E, Shariv I, Eisenstein M, Friesem AA, Aflalo C, Vakser IA. Molecular surface recognition: determination of geometric fit between proteins and their ligands by correlation techniques. Proc Natl Acad Sci U S A. 1992;89:2195–9. 1549581 10.1073/pnas.89.6.2195 PMC48623

[20] Lobb RJ, Becker M, Wen SW, Wong CS, Wiegmans AP, Leimgruber A, Moller A. Optimized exosome isolation protocol for cell culture supernatant and human plasma. J Extracell Vesicles. 2015;4:27031. 26194179 10.3402/jev.v4.27031 PMC4507751

[21] Hu Q, Yao J, Wu X, Li J, Li G, Tang W, Liu J, Wan M. Emodin attenuates severe acute pancreatitis-associated acute lung injury by suppressing pancreatic exosome-mediated alveolar macrophage activation. Acta Pharm Sin B. 2022;12:3986–4003. 36213542 10.1016/j.apsb.2021.10.008 PMC9532455

[22] Shen K, Wang X, Wang Y, Jia Y, Zhang Y, Wang K, Luo L, Cai W, Li J, Li S, et al. miR-125b-5p in adipose derived stem cells exosome alleviates pulmonary microvascular endothelial cells ferroptosis via Keap1/Nrf2/GPX4 in sepsis lung injury. Redox Biol. 2023;62:102655. 36913799 10.1016/j.redox.2023.102655 PMC10023991

[23] Shaikh TB, Kuncha M, Andugulapati SB, Sistla R. Dehydrozingerone alleviates pulmonary fibrosis via Inhibition of inflammation and epithelial-mesenchymal transition by regulating the Wnt/beta-catenin pathway. Eur J Pharmacol. 2023;953:175820. 37245857 10.1016/j.ejphar.2023.175820

[24] Xiong YC, Chen XY, Yang XD, Zhang H, Li XM, Wang ZL, Feng SZ, Wen W, Xiong XQ. MiRNA transcriptomics analysis shows miR-483-5p and miR-503-5p targeted MiRNA in extracellular vesicles from severe acute pancreatitis-associated lung injury patients. Int Immunopharmacol. 2023;125:111075. 37864909 10.1016/j.intimp.2023.111075

[25] Zhang Y, Gu C, Zhao L, Wang B, Sun Y, Lou Y, Ma D, Wang Y. Obesity-associated reduction of miR-150-5p in extracellular vesicles promotes ventilator-induced lung injury by modulating the lysosomal degradation of VE-cadherin. Cell Death Discov. 2025;11:220. 40328745 10.1038/s41420-025-02499-5 PMC12055972

[26] Younes N, Zhou L, Amatullah H, Mei SHJ, Herrero R, Lorente JA, Stewart DJ, Marsden P, Liles WC, Hu PZ, dos Santos CC. Mesenchymal stromal/stem cells modulate response to experimental sepsis-induced lung injury via regulation of miR-27a-5p in recipient mice. Thorax. 2020;75:556–67. 32546573 10.1136/thoraxjnl-2019-213561 PMC7361025

[27] Kobeissy PH, Deneve-Larrazet C, Marvaud JC, Kansau I. MicroRNA miR-27a-5p reduces intestinal inflammation induced by clostridioides difficile flagella by regulating the NF-kappaB signaling pathway. J Infect Dis. 2024;231:e38–46. 10.1093/infdis/jiae396 PMC11793073 39126324

[28] Kanne JP, Little BP, Schulte JJ, Haramati A, Haramati LB. Long-term lung abnormalities associated with COVID-19 pneumonia. Radiology. 2023;306:e221806. 36040336 10.1148/radiol.221806 PMC9462591

[29] Agrawal DK, Smith BJ, Sottile PD, Albers DJ. A Damaged-Informed lung ventilator model for ventilator waveforms. Front Physiol. 2021;12:724046. 34658911 10.3389/fphys.2021.724046 PMC8517122

[30] Liu H, Gu C, Liu M, Liu G, Wang D, Liu X, Wang Y. Ventilator-induced lung injury is alleviated by inhibiting NLRP3 inflammasome activation. Mol Immunol. 2019;111:1–10. 30952009 10.1016/j.molimm.2019.03.011

[31] Birdsall V, Kirwan K, Zhu M, Imoto Y, Wilson SM, Watanabe S, Waites CL. Axonal transport of Hrs is activity dependent and facilitates synaptic vesicle protein degradation. Life Sci Alliance. 2022;5:e202000745. 10.26508/lsa.202000745 PMC9152131 35636965

[32] Walter F, Schmid J, Dussmann H, Concannon CG, Prehn JH. Imaging of single cell responses to ER stress indicates that the relative dynamics of IRE1/XBP1 and PERK/ATF4 signalling rather than a switch between signalling branches determine cell survival. Cell Death Differ. 2015;22:1502–16. 25633195 10.1038/cdd.2014.241 PMC4532775

[33] Luo X, Alfason L, Wei M, Wu S, Kasim V. Spliced or unspliced, that is the question: the biological roles of XBP1 isoforms in pathophysiology. Int J Mol Sci. 2022;23:2746. 35269888 10.3390/ijms23052746 PMC8910952

[34] Chalmers FE, Mogre S, Rimal B, Son J, Patterson AD, Stairs DB, Glick AB. The unfolded protein response gene Ire1alpha is required for tissue renewal and normal differentiation in the mouse tongue and esophagus. Dev Biol. 2022;492:59–70. 36179879 10.1016/j.ydbio.2022.09.009

[35] Gao M, Yu T, Liu D, Shi Y, Yang P, Zhang J, Wang J, Liu Y, Zhang X. Sepsis plasma-derived Exosomal miR-1-3p induces endothelial cell dysfunction by targeting SERP1. Clin Sci (Lond). 2021;135:347–65. 33416075 10.1042/CS20200573 PMC7843403

[36] Wu SW, Peng CK, Wu SY, Wang Y, Yang SS, Tang SE, Huang KL. Obesity attenuates Ventilator-Induced lung injury by modulating the STAT3-SOCS3 pathway. Front Immunol. 2021;12:720844. 34489970 10.3389/fimmu.2021.720844 PMC8417798

[37] Ni YN, Luo J, Yu H, Wang YW, Hu YH, Liu D, Liang BM, Liang ZA. Can body mass index predict clinical outcomes for patients with acute lung injury/acute respiratory distress syndrome? A meta-analysis. Crit Care. 2017;21:36. 28222804 10.1186/s13054-017-1615-3 PMC5320793

[38] Palma G, Sorice GP, Genchi VA, Giordano F, Caccioppoli C, D’Oria R, Marrano N, Biondi G, Giorgino F, Perrini S. Adipose tissue inflammation and pulmonary dysfunction in obesity. Int J Mol Sci. 2022;23:7349. 10.3390/ijms23137349 PMC9267094 35806353

