# Supplementary material for: MiR-27a-5p deficiency in plasma exosomes derived from obese individuals exacerbates ventilator-induced lung injury in vitro and in vivo
Source: J Transl Med. 2025 Nov 11;23:1264. doi: 10.1186/s12967-025-06988-0 (PMC12607151; doi:10.1186/s12967-025-06988-0)
Supplement: Supplementary file 1 — Supplementary Material 1 [file 12967_2025_6988_MOESM1_ESM.docx]

**Online supplement for Wang B, et al. MiR-27a-5p Deficiency in Plasma Exosomes Derived from Obese Individuals Exacerbates Ventilator-Induced Lung Injury in vitro and in vivo**

**Supplemental methods (from page 1)**

**Supplemental tables 1-8 (from page 7)**

**Supplemental figures (from page 19)**

**Supplemental Methods**

**Bioinformatics analysis**

A cell model of VILI was established by cyclic stretching of HPMECs, followed by transcriptome analysis using an Affymetrix Human Transcriptome Array 2.0. The microarray data has been deposited into a public domain (accession: GSE166772). A mechanically stretched sample of A549 cells was obtained from the gene expression profile of GSE1541. Subsequently, the results from the dataset were evaluated and analyzed using R software.

**In vitro experiments**

***Cells***

MLE-12 and BEAS-2B cells were purchased from Shanghai Zhong Qiao Xin Zhou Biotechnology (Shanghai, China) and 293T cells was purchased from Servicebio company (Wuhan, China).

***Cell culture and cyclic stretching***

The lung cell line MLE-12 was cultured in DMEM/F-12 medium supplemented with 10% fetal bovine serum. The bronchial epithelial cell lines BEAS-2B and 293T were cultured in high-glucose DMEM supplemented with 10% fetal bovine serum. Cultured MLE-12 or BEAS-2B cells were seeded on type I collagen-coated Flexcell stretch plates. At 90% confluence, these plates were placed in a cell stretch stress loading system (Flexcell, North Carolina, USA) at 0.5 Hz and 20% stretch with a stretch relaxation ratio of 1:1.

***Transfection***

When the cell density reached 70%, MLE-12 cells and BEAS-2B cells were transfected with 0.02 OD/ml of si-Pyk2 or miR-27a-5p with Lipofectamine 3000. After 6 h, the medium was replaced with original medium and the transfected cells were used for further experiments after 48 h. The transfection efficiency was verified by western blot analysis.

***Immunofluorescence staining***

After stretching, the BEAS-2B cells were fixed with 4% paraformaldehyde and blocked with 5% BSA, followed by incubation with an anti-E-cadherin antibody (1:300) overnight at 4 °C. Subsequently, the cells were washed three times in PBST and incubated with a secondary antibody (1 h). Nuclei were counterstained with 4′,6-diamidino-2-phenyl indole dihydrochloride (2 μg/ml) for 10 min and then rinsed three times with PBS. The Flex film was removed with a razor blade, and the cells were mounted on a slide. The E-cadherin and lysosome colocalization was imaged with a laser confocal microscopy and with ImageJ software. All antibodies used in this and other part are listed in Table S1 (see below).

***Western blotting and immunoprecipitation***

MLE-12 cells and lung tissue were lysed in RIPA buffer supplemented with 1 mM protease inhibitor, 1 mM sodium orthovanadate, and 1 mM PMSF. After ultrasonication, the protein concentration in the supernatant was measured using a BCA protein concentration determination kit. Proteins were separated on a 10% acrylamide gel by SDS‒PAGE and transferred to a PVDF membrane. The membrane was blocked in 5% BSA at room temperature for 1 h. The primary antibody was then applied to the membrane at 4 °C overnight. After washing with TBST, the membrane was incubated with a secondary antibody at room temperature for 1 h. Protein bands were detected by chemiluminescent HRP substrate The relative band densities of various proteins were analyzed with ImageJ software.

After centrifugation of the MLE-12 cell lysate, the supernatant was incubated with 1 mg of normal IgG and protein A/G plus-agarose beads for 30 min for reclarification. Then, the supernatant was incubated with Pyk2 and Hgs antibodies and 20 μl of protein A/G Plus-agarose beads overnight in an oscillator. The immunoprecipitants were dissolved in 20 μl of SDS‒PAGE sample buffer for western blot analysis.

***RNA isolation and RT‒PCR***

Total RNA was extracted from lung tissue or cultured cells using the Fastagen RNA extraction kit (Shanghai, China), and cDNA was synthesized using the PrimeScript™ RT reagent Kit (TaKaRa, Tokyo, Japan), according to the manufacturer's instructions. Subsequent RT-qPCR analysis was performed on a Light Cycler instrument (Bio-Rad, California, USA), employing the FastStart Essential DNA Green Master Kit (Roche, Basel, Switzerland). The expression data were normalized relative to the mRNA levels of GAPDH or the miRNA U6. Each sample was loaded in triplicate for analysis, and the results were evaluated using the 2−ΔΔCt method. The sequences of the primers used are listed in Table S2 (see below).

***Dual luciferase reporter assay***

293T cells were seeded in triplicate into a 96-well plate at 70% confluence and transfected with luciferase reporter and transcription factor plasmids using Lipofectamine 2000 after 24 h culture in accordance with the manufacturer’s instructions. Firefly luciferase and Renilla luciferase signals were detected at 48 h after transfection using a dual luciferase reporting kit. Relative promoter activity is expressed as the ratio of firefly to Renilla luciferase activity. The sequences of the Pyk2 promoter and mutants driving luciferase expression are listed in Table S3 (see below).

***Transmission electron microscopy***

After fixation, rinsing, dehydration, infiltration, embedding and sectioning, the processed MLE-12 cells were subjected to transmission electron microscopy (TEM) (HT-7800, Hitachi, Japan) assessment.

***Molecular docking***

Both the Hgs (3ZYQ) and Pyk2 (3CC6) crystal structures were reported in the Protein Data Bank database (https://www.rcsb.org/). The Docking Web Server (GRAMM) (https://gramm.compbio.ku.edu/) was used for molecular docking. The Hgs and Pyk2 interaction diagram was generated through PyMOL.

***Exosome isolation and characterization***

Extracellular vesicles were extracted from blood samples collected clinically using ultracentrifugation. The extracted exocrine body weight was suspended in PBS and stored at -80 °C for further use. The morphology of the isolated exosomes was observed by transmission electron microscopy (TEM). The particle size distribution and concentration of extracellular vesicles were analyzed using nanoparticle tracking analysis (NTA). The protein biomarkers CD9, CD63, and TSG101 were extracted from exosomes through western blotting detection. The extracted plasma exosomes were labeled with PKH67 (Beijing Fluorescence Biotechnology Co., Ltd., PKH67), added to MLE-12 cells for 12 hours, and observed via laser confocal microscopy.

***Exosomes uptake assay and co-culture***

The process began with resuspending the extracellular vesicles in 200 μL of diluent C. Concurrently, the PKH67 labeling solution was diluted with diluent C in a ratio ranging from 100:1 to 200 μL. Following this, the two solutions are evenly mixed and incubated at 37 °C for a duration of 5 minutes. Subsequently, an equal volume of 5% BSA was introduced to the mixture, which was then centrifuged at a speed of 120000 × g for 70 minutes at 4 °C. Post-centrifugation, the supernatant was carefully removed, and the extracellular vesicles were resuspended in PBS before their incorporation into the cell culture medium.

Subsequently, MLE-12 cells were co-cultured with the aforementioned mixture for a period of 12 hours. Upon completion of the cultivation, the cell nuclei were stained with DAPI, while the cytoplasm was labeled using an F-actin staining agent. Ultimately, a micrograph was captured under a fluorescence microscope to document the fluorescence image of the cells.

MLE-12 cells were cultured in the presence of extracellular vesicles, which were derived from both normal controls and obese patients at a concentration of 20 μg/mL, for a period of 24 hours [17]. Following this incubation, the cells were subjected to cyclic stretching at a frequency of 0.5 Hz with a 20% strain, employing a stretching relaxation ratio of 1:1.

**In vivo experiments**

***Animals***

C57BL/6 mice (Male, 5-6 weeks old) were obtained from Beijing Vital River Laboratory Animal Technology (Beijing, China).

***Liposome preparation and in vivo gene delivery***

Preparation and transfection of liposomes before mechanical ventilation in mice. si-Pyk2 or miR-27a-5p at an OD of 1.5 was dissolved in 50 µL of DEPC water. Then, 25 μL of Entranster™ in vivo was diluted in 25 μL of DEPC water, and 10% glucose was added to the diluted si-Pyk2 or miR-27a-5p and transfection reagent to a final glucose concentration of 5%. The reagents were mixed at room temperature and left to stand for 15 minutes before use. Mice received injections of the prepared liposome solutions via the tail vein. Two days later, si-Pyk2 or miR-27a-5p at 1.5 OD was transfected into the mice via the same method. After 2 days, the mice were subjected to experiments. The transfection efficiency of si-Pyk2 was verified with western blot analysis of lung homogenates. The sequences of the miRNAs and siRNAs used are listed in Table S4 (see below). One hundred micrograms of plasma exosomes were injected into mice before mechanical ventilation [18].

***Experimental protocol***

Mice were randomly divided into 13 experimental groups (n = 6/group) (see Table S5 below). Mice in the Pyk2-knockdown group were transfected with si-Pyk2 for 2 days. Mice were anesthetized by an intraperitoneal injection of 60 mg/kg pentobarbital sodium. They then underwent tracheotomy for mechanical ventilation with a high tidal volume ventilation mode. The ventilation parameters were set as follows: tidal volume 28 ml/kg, respiratory rate 60 times/min, I/E ratio of 1:2, 0 cm H2O end-expiratory pressure, and fraction of inspired oxygen 21%. The control and Pyk2 knockdown groups did not receive mechanical ventilation. The experiments were conducted on a heat lamp to maintain the temperature at 37 °C. Lung function was tested after 4 h of mechanical ventilation, after which the mice were euthanized. Lung injury was assessed by analyzing proinflammatory factors in BALF, the W/D ratio, HE staining, and E-cadherin expression in lung tissue.

***BALF collection***

After treatment, 0.3 mL of cold saline was injected into the lungs of the mice through the trachea three times with a 1 mL syringe. After a 3 s interval, the saline was slowly withdrawn and stored at -80 °C.

***ELISA***

The collected BALF was centrifuged at 1000 × g for 20 minutes at 4 ℃, and the supernatant was taken for ELISA detection of IL-1 β, TNF - α, and IL- 6.

***HE staining***

After pretreatment, fresh left lung tissues were collected and fixed in 4% paraformaldehyde. After dehydration, the samples were embedded in paraffin, sectioned, dewaxed, stained and dehydrated. Images were captured under a microscope with a camera (Nikon, Tokyo, Japan).

***Measurement of the W/D ratio of lung tissue***

After treatment, the right lung tissue was removed quickly. Blood on the lung tissue surface was removed with cold PBS, followed by drying with filter paper. The wet weight of the lung tissue was measured. The lung tissue was then dried in an oven to a constant weight (60 °C for 48 h). The dry lung weight was recorded, and the W/D ratio was calculated.

***Lung function test***

At the end of experiments, the mice with tracheal intubation were quickly connected to the pulmonary function instrument (FlexiVent system, Montreal, Canada). The parameters of the pulmonary function instrument were set as a respiratory rate of 150 times per minute, a tidal volume of 10 mL per kilogram of body weight, and a positive end-expiratory pressure maintained at 3 cmH2O. Subsequently, the data on static lung compliance (Cst), elastance (Ers), and tissue damping (G) were measured and recorded as reported previously [19].

***Immunohistochemical staining***

After the antigen retrieval of the wax tissue section through, they were then blocked with 1% BSA followed by primary antibodies and then corresponding secondary antibodies (the primary antibody and Opal fluorophores paired as: anti SFTPC/Opal 480, anti Pyk2/Opal 570, anti E-cadherin/Opal 620, anti IL1 β/ Opal 690, and anti-CD3/Opal 780). After DAPI applied, the sections were sealed with a sealing agent and the micrographs were taken with a fluorescent microscope for further data analyses.

**Human blood samples**

***Inclusion and exclusion criteria***

All patients and healthy controls participating in the study signed written informed consent forms before recruitments. Inclusion criteria: Obese individuals (body mass index (BMI) > 30 kg m^-2^) at 18 - 60 years old were recruited from patients undergoing weight loss surgery at the First Affiliated Hospital of Shandong First Medical University from April to June 2023. Healthy non obese subjects (BMI<25 kg m^-2^) were matched with age, gender, and race of patients. Exclusion criteria included a history of lung diseases such as obstructive airway disease (asthma or chronic obstructive pulmonary disease); smoked in the past 6 months or smoking history>20 years; respiratory infections or pregnancy; mechanical ventilation within 30 days; lung surgery or other surgical procedures.

The blood samples were obtained from six patients (3 males and females) at the First Affiliated Hospital of Shandong First Medical University from April to June 2023 and six healthy volunteers (3 males and females). Within two hours post blood collection, exosomes are extracted individually and promptly stored at a temperature of -80 °C. The baseline characteristics of the study population are shown in Table S6 (see below).

***Microarray***

Exosomes were dispatched to Genesky Biotechnology Co. Ltd. (Shanghai, China), for miRNA sequencing. The total RNA extracted from these exosomes underwent concentration determination prior to being subjected to next-generation sequencing on the Illumina MiSeq platform. Following data preprocessing, differentially expressed miRNAs were identified, utilizing thresholds of an absolute log2FC > 1 and a p-value < 0.05.

**Statistical analyses**

All results are expressed as the mean ± standard deviation (SD). One-way or two-way analysis of variance (ANOVA) followed by post hoc Newman-Keuls test for multi-comparison or Student’s t test was used for between two groups as appropriate with GraphPad software. A p value less than 0.05 was considered to be of a statistical significance.

**Supplemental tables**

**Table S1. Suppliers of antibodies used**

| Antibody name | Company | Catalog number | Source | Reactive | Dilution |
| --- | --- | --- | --- | --- | --- |
| CD63 | Santa Cruz | sc-5275 | Mouse | M, H, R | WB 1:200 |
| CD9 | Santa Cruz | sc-13118 | Mouse | M, H, R | WB 1:500 |
| TSG101 | Santa Cruz | sc-7964 | Mouse | M, H, R | WB 1:500 |
| ALIX | Servicebio | GB124080 | Mouse | M, H, R | WB 1:1000 |
| Calnexin | Santa Cruz | sc-23954 | Mouse | M, H, R | WB 1:200 |
| Pyk2 | Proteintech | 17592-1-AP | Rabbit | M, H, R | WB 1:1000 |
| Pyk2 | Abcam | Ab32571 | Rabbit | M, H, R | WB 1:1000 |
| p-Pyk2 | CST | #3291 | Rabbit | M, H | WB 1:1000 |
| Hgs | Abcam | ab155539 | Rabbit | M, H, R | WB 1:1000 |
| GAPDH | Abcam | ab181602 | Rabbit | M, H, R | WB 1:10000 |
| GAPDH | Servicebio | GB15004 | Rabbit | M, H, R | WB 1:6000 |
| GAPDH | CST | #2118 | Rabbit | M, H, R | WB 1:1000 |
| NaK-ATPase | Proteintech | 144181-1-1P | Rabbit | M, H, R | WB 1:5000 |
| NaK-ATPase | CST | #3010 | Rabbit | M, H, R | WB 1:1000 |
| E-cadherin | CST | #14472 | Mouse | M, H, R | WB 1:1000 |
| E-cadherin | Abcam | ab76319 | Rabbit | M, H, R | WB 1:200 |
| Grp78 | CST | #3177 | Rabbit | M, H | WB 1:1000 |
| Xbp1s | CST | #12782 | Rabbit | H | WB 1:1000 |
| Xbp1s | Proteintech | 24868-1-AP | Rabbit | M, H, R | WB 1:1000 |
| E-cadherin | CST | #3195 | Rabbit | M, H | IF 1:400 |
| LAMP1 | Santa Cruz | sc-20011 | Mouse | M, H, R | IF 1:50 |
| E-cadherin | Santa Cruz | sc-8426 | Mouse | M, H, R | IHC1:200 |
| Pyk2 | Abcam | Ab32571 | Rabbit | M, H, R | IHC 1:1200 |
| SFTPC | Abcam | Ab211326 | Rabbit | M | IHC 1:200 |
| IL-1β | Abcam | Ab283818 | Rabbit | M, H, R | IHC 1:200 |
| CD3 | Abcam | Ab16669 | Rabbit | M, H, R | IHC 1:200 |

**Table S2. Primer sequence for RT-PCR**

| Targets | Forward (from 5’ to 3’) | Reverse (from 5’ to 3’) |
| --- | --- | --- |
| Homo-β-actin | GTATCCTGACCCTGAAGTACC | GAAGGTCTCAAACATGATCT |
| Homo-Pyk2 | GTCTTCAAACGCCACAGCAT | CTGGCGTCAATGGGGACTTATC |
| Homo-Hgs | TGATGACCCGTAAGCACCAC | CTTTCCCTCCGCTTTCCTGT |
| Homo-Xbp1 | GTCCGCAGCACTCAGACTAC | CTCTGGGGAAGGGCATTTGA |
| Mus-GAPDH | TGCCCAGAACATCATCCCT | GGTCCTCAGTGTAGCCCAAG |
| Mus-Pyk2 | TGGGACACTACCTGGAACGA | CCTCAATGTACCGGGAGAGC |
| Mus-Hgs | CCTCCACAGACCAGCAACAT | CGGTCATGAGATTCTGCATATTGTA |

**Table S3. Sequences of the Pyk2 promoter and mutants driving luciferase expression**

| Targets | Sequences (from 5’ to 3’) | Site |
| --- | --- | --- |
| HOMO-PYK2 WT | GGGGACACGTCGGG | 1732-1745 |
| HOMO-PYK2 MUT | CCACGACAAATCAA | 1732-1745 |

**Table S4. miRNA and siRNA sequences**

| Targets | sense (from 5’ to 3’) | antisense (from 5’ to 3’) |
| --- | --- | --- |
| Negative control | UUCUUCGAACGUGUCACGUTT | ACGUGACACGUUCGGAGAATT |
| Homo-si-Hgs | GGACCUGCUGAAGAGACAATT | GCCUGUACUCUUCACCUGUTT |
| Homo-si-Xbp1 | GCUUGGUGUAAACCAUUCUTT | AGAAUGGUUUACACCAAGCTT |
| Mus-si-Pyk2 | CUACCUGGAACGAAAUAAATT | UUUAUUUCGUUCCAGGUAGTT |
| Mus-si-Hgs | AUCUGUAGAAUGGACUCCCTT | ACAUUAACUUCCACUUGCCTT |
| Mus-si-Xbp1 | CAAGCUGGAAGCCAUUAAUTT | AUUAAUGGCUUCCAGCUUGTT |
| Mimic-miR-27a-5p | AGGGCUUAGCUGCUUGUGAGCA | CUCACAAGCAGCUAAGCCCUUU |

**Table S5. Grouping of mice**

| Group | Number of mice | Corresponding figures |
| --- | --- | --- |
| MV 0h | 6 | Figure 1 and S1 |
| MV 2h | 6 | Figure 1 and S1 |
| MV 4h | 6 | Figure 1 and S1 |
| si-NC | 6 | Figure S1 |
| si-Pyk2 | 6 | Figure S1 |
| MV 0h + si-NC | 6 | Figure3 |
| MV 4h + si-NC | 6 | Figure3 |
| MV 0h + si-Pyk2 | 6 | Figure3 |
| MV 4h + si-Pyk2 | 6 | Figure3 |
| MV 0h + Control-exo | 6 | Figure8 |
| MV 4h + Control-exo | 6 | Figure8 |
| MV 4h + Obesity-exo | 6 | Figure8 |
| MV 4h + Obesity-exo + miR-27a-5p | 6 | Figure8 |

**Table S6.** **The baseline characteristics of Obese patients and healthy controls**

| Baseline characteristics | Obesity (n=6) | Control (n=6) | *P* |
| --- | --- | --- | --- |
| Male | 3 | 3 |  |
| Female | 3 | 3 |  |
| Age, yr | 26 (22-32) | 26 (25-30) | *p*> 0.05 |
| Weight, kg | 130 (24) | 61 (6) | *P*< 0.05 |
| BMI, kg m^-2^ | 44 (5) | 22 (1) | *P<* 0.05 |
| Surgical history | 0 | 0 |  |
| Pulmonary diseases | 0 | 0 |  |
| Smoking history | 0 | 0 |  |
| Respiratory infection within 3 months | 0 | 0 |  |
| History of MV within 30 days | 0 | 0 |  |

Quantitative data is expressed as mean (SD) or median (range). Student's *t*-test is used for continuous variable analysis (age, weight, BMI), while chi square test is used for categorical variable analysis (gender). Statistical analysis was conducted using GraphPad software.

**Table S7. DEGs of mechanical stretching HPMECs**

| Gene sample | logFC | AveExpr | t | P.Value | adj.P.Val | B |
| --- | --- | --- | --- | --- | --- | --- |
| LOC344887 | 1.108801 | 4.494957 | 9.471538 | 3.27E-05 | 0.99995 | -3.31064 |
| LPXN | 0.578278 | 4.954725 | 5.853683 | 0.000653 | 0.99995 | -3.49434 |
| HERC2P4 | -0.59188 | 6.489008 | -5.58691 | 0.000858 | 0.99995 | -3.5193 |
| FOXF1 | 0.555858 | 5.022173 | 5.523248 | 0.000917 | 0.99995 | -3.52564 |
| STC1 | 1.267582 | 5.70774 | 5.180604 | 0.001323 | 0.99995 | -3.56257 |
| BHLHE40 | 0.736535 | 6.525604 | 5.017922 | 0.001583 | 0.99995 | -3.58191 |
| EID3 | 0.587094 | 6.248527 | 4.634381 | 0.002453 | 0.99995 | -3.63276 |
| HERC2P9 | -0.609 | 7.507977 | -4.55574 | 0.00269 | 0.99995 | -3.64419 |
| OTTHUMG00  000074396 | -0.64969 | 2.87506 | -4.49041 | 0.002907 | 0.99995 | -3.65396 |
| ZNF175 | 0.647837 | 5.849982 | 4.442168 | 0.003079 | 0.99995 | -3.66134 |
| HERC2P2 | -0.59678 | 7.58515 | -4.41132 | 0.003195 | 0.99995 | -3.66613 |
| DOCK10 | 0.686155 | 6.287747 | 4.405183 | 0.003219 | 0.99995 | -3.66709 |
| SESN3 | -0.68586 | 4.759354 | -4.32368 | 0.003552 | 0.99995 | -3.68008 |
| TGFB2 | -0.55538 | 5.894709 | -4.31223 | 0.003602 | 0.99995 | -3.68194 |
| HERC2P3 | -0.53452 | 7.478831 | -4.12436 | 0.004539 | 0.99995 | -3.71371 |
| MIR21 | 0.54187 | 5.84159 | 4.034963 | 0.005076 | 0.99995 | -3.72969 |
| ADRB2 | 0.507929 | 4.66429 | 3.978529 | 0.005451 | 0.99995 | -3.74008 |
| MPP4 | 0.826707 | 5.36359 | 3.873725 | 0.00623 | 0.99995 | -3.76 |
| SSH1 | 0.607915 | 6.491223 | 3.810329 | 0.00676 | 0.99995 | -3.77246 |
| RPA4 | 0.538324 | 2.93615 | 3.764097 | 0.007177 | 0.99995 | -3.78175 |
| OTTHUMG00  000015537 | 0.791967 | 3.961816 | 3.739583 | 0.007409 | 0.99995 | -3.78675 |
| ZC3H12C | 0.957078 | 5.962516 | 3.569538 | 0.009266 | 0.99995 | -3.82277 |
| TRDV1 | -0.56195 | 3.306574 | -3.54859 | 0.009528 | 0.99995 | -3.82737 |
| BGN | -0.65929 | 8.14475 | -3.50438 | 0.010107 | 0.99995 | -3.83722 |
| HMOX1 | 0.723912 | 6.758448 | 3.437726 | 0.011053 | 0.99995 | -3.85238 |
| FERMT3 | 0.524033 | 6.545242 | 3.424743 | 0.011248 | 0.99995 | -3.85538 |
| PTGS2 | 1.063733 | 4.844336 | 3.40721 | 0.011517 | 0.99995 | -3.85945 |
| HBEGF | 0.57518 | 6.469102 | 3.39315 | 0.011738 | 0.99995 | -3.86274 |
| POSTN | -0.84484 | 5.967029 | -3.38699 | 0.011837 | 0.99995 | -3.86418 |
| IL1B | 0.618245 | 4.661543 | 3.303171 | 0.013266 | 0.99995 | -3.88419 |
| CNKSR3 | 0.632713 | 5.466657 | 3.270913 | 0.013865 | 0.99995 | -3.89207 |
| NEDD9 | 0.732431 | 5.500585 | 3.191356 | 0.015468 | 0.99995 | -3.91189 |
| IL1A | 1.2704 | 3.44631 | 3.065883 | 0.018415 | 0.99995 | -3.94436 |
| PTGR1 | 0.812419 | 6.49834 | 3.031113 | 0.019333 | 0.99995 | -3.95362 |
| MGP | -0.54974 | 7.246686 | -3.01922 | 0.019658 | 0.99995 | -3.95681 |
| ITGA11 | 0.581854 | 6.107655 | 2.908063 | 0.022995 | 0.99995 | -3.98732 |
| MIR29A | 0.919542 | 2.250236 | 2.84405 | 0.025184 | 0.99995 | -4.00542 |
| MGARP | -0.80809 | 5.806285 | -2.7502 | 0.028798 | 0.99995 | -4.03266 |
| NAV3 | 0.902739 | 5.786729 | 2.736329 | 0.029377 | 0.99995 | -4.03676 |
| NQO1 | 0.91003 | 8.437465 | 2.710477 | 0.030488 | 0.99995 | -4.04445 |
| RND1 | 0.632016 | 5.333594 | 2.636763 | 0.033906 | 0.99995 | -4.06671 |
| NFKBIZ | 0.864231 | 5.105445 | 2.510913 | 0.040693 | 0.99995 | -4.10587 |
| DHCR7 | 0.520518 | 7.866052 | 2.48969 | 0.04197 | 0.99995 | -4.11262 |
| GCLM | 0.552854 | 6.527249 | 2.486624 | 0.042158 | 0.99995 | -4.11359 |
| SNORD12 | 0.563885 | 6.127039 | 2.469369 | 0.043231 | 0.99995 | -4.11911 |
| ASAP1-IT1 | 0.535845 | 4.648949 | 2.400209 | 0.047824 | 0.99995 | -4.14149 |
| IL8 | 1.36001 | 5.247816 | 2.375668 | 0.049571 | 0.99995 | -4.14952 |

**Table S8. DEGs of mechanical stretching A549**

| Gene sample | logFC | AveExpr | t | P.Value | adj.P.Val | B |
| --- | --- | --- | --- | --- | --- | --- |
| MUC4 | 3.048768 | 2.312672 | 8.255615 | 0.000498 | 0.99967 | -4.49348 |
| KLRA1P | 2.815804 | 2.382858 | 7.560240 | 0.000743 | 0.99967 | -4.49509 |
| GADD45G | 2.784392 | 2.814298 | 7.216974 | 0.000915 | 0.99967 | -4.49603 |
| FOS | -3.824171 | 4.526449 | -6.891669 | 0.001125 | 0.99967 | -4.49705 |
| GH2 | -2.417769 | 2.520414 | -6.771876 | 0.001216 | 0.99967 | -4.49745 |
| CYP4B1 | 2.361346 | 2.840274 | 6.588701 | 0.001373 | 0.99967 | -4.49811 |
| CRB1 | 2.258733 | 3.982945 | 6.297066 | 0.001676 | 0.99967 | -4.49926 |
| ING4 | 2.329883 | 4.361242 | 6.106641 | 0.001917 | 0.99967 | -4.50009 |
| KLF6 | -2.144429 | 6.001363 | -5.967679 | 0.002119 | 0.99967 | -4.50074 |
| GPC5 | 2.172854 | 2.533311 | 5.947696 | 0.002150 | 0.99967 | -4.50083 |
| CXCR3 | -2.169620 | 3.829597 | -5.943339 | 0.002157 | 0.99967 | -4.50086 |
| SCARF1 | 2.327005 | 2.035300 | 5.816817 | 0.002368 | 0.99967 | -4.50149 |
| DLG3 | -2.151654 | 3.649688 | -5.805881 | 0.002387 | 0.99967 | -4.50154 |
| JAK2 | -2.192760 | 2.144848 | -5.691732 | 0.002600 | 0.99967 | -4.50214 |
| KIF25 | 2.400914 | 2.506692 | 5.674385 | 0.002634 | 0.99967 | -4.50224 |
| FABP4 | -2.241730 | 3.228920 | -5.602275 | 0.002783 | 0.99967 | -4.50263 |
| STMN2 | -2.059510 | 2.213608 | -5.601067 | 0.002785 | 0.99967 | -4.50264 |
| SLC30A4 | -1.986414 | 3.356391 | -5.582794 | 0.002825 | 0.99967 | -4.50274 |
| CD1B | -2.212543 | 3.361194 | -5.571247 | 0.002850 | 0.99967 | -4.50281 |
| DLEU2 | 2.105982 | 3.298302 | 5.536718 | 0.002927 | 0.99967 | -4.50301 |
| PTPN22 | -2.371157 | 2.664560 | -5.462357 | 0.003100 | 0.99967 | -4.50344 |
| HAO1 | -1.936056 | 3.215922 | -5.391729 | 0.003277 | 0.99967 | -4.50387 |
| F2 | 2.094962 | 3.474614 | 5.308049 | 0.003501 | 0.99967 | -4.50440 |
| SLURP1 | 1.884652 | 3.724337 | 5.293554 | 0.003542 | 0.99967 | -4.50449 |
| PRMT8 | 2.434110 | 2.968557 | 5.265807 | 0.003621 | 0.99967 | -4.50467 |
| PRKCA | -2.323811 | 3.425182 | -5.166235 | 0.003924 | 0.99967 | -4.50533 |
| MEOX1 | -2.366898 | 2.735957 | -5.073025 | 0.004235 | 0.99967 | -4.50598 |
| DELEC1 | -1.956396 | 3.261636 | -5.060484 | 0.004279 | 0.99967 | -4.50607 |
| WT1-AS | -2.032282 | 3.491238 | -5.042097 | 0.004345 | 0.99967 | -4.50621 |
| EN1 | 2.137079 | 2.891459 | 5.039305 | 0.004355 | 0.99967 | -4.50623 |
| LPAL2 | 2.590555 | 2.855288 | 5.033847 | 0.004375 | 0.99967 | -4.50627 |
| BGN | 1.925872 | 3.059615 | 4.988765 | 0.004542 | 0.99967 | -4.50660 |
| CX3CR1 | 2.405140 | 2.938862 | 4.971506 | 0.004608 | 0.99967 | -4.50672 |
| KNG1 | -2.251817 | 3.226606 | -4.953155 | 0.004679 | 0.99967 | -4.50686 |
| SLC13A1 | -1.759273 | 1.375322 | -4.940732 | 0.004728 | 0.99967 | -4.50696 |
| PTX3 | 1.899618 | 2.514286 | 4.936866 | 0.004743 | 0.99967 | -4.50698 |
| MTHFR | 1.872126 | 4.431272 | 4.912575 | 0.004841 | 0.99967 | -4.50717 |
| KRTAP9-9 | -1.793887 | 2.461717 | -4.894807 | 0.004915 | 0.99967 | -4.50731 |
| RFPL1 | -1.929758 | 1.934728 | -4.866742 | 0.005033 | 0.99967 | -4.50753 |
| NTF3 | 1.791697 | 4.128867 | 4.852555 | 0.005094 | 0.99967 | -4.50764 |
| RAI2 | -2.162044 | 3.027673 | -4.836708 | 0.005163 | 0.99967 | -4.50776 |
| ASPN | -1.960754 | 2.295521 | -4.814569 | 0.005262 | 0.99967 | -4.50794 |
| RAB9BP1 | -2.090673 | 2.175008 | -4.773769 | 0.005449 | 0.99967 | -4.50827 |
| ZNF345 | 1.787698 | 2.222658 | 4.681171 | 0.005904 | 0.99967 | -4.50905 |
| RARG | -1.703406 | 4.767680 | -4.633870 | 0.006154 | 0.99967 | -4.50946 |
| HP | 1.969916 | 3.825990 | 4.631842 | 0.006165 | 0.99967 | -4.50948 |
| SCN2B | 2.553487 | 3.246267 | 4.572153 | 0.006499 | 0.99967 | -4.51001 |
| EFS | 1.661558 | 2.649143 | 4.491870 | 0.006981 | 0.99967 | -4.51075 |
| CRHBP | -2.770096 | 3.351575 | -4.477628 | 0.007071 | 0.99967 | -4.51088 |
| ENPP1 | -1.701492 | 3.143601 | -4.428662 | 0.007391 | 0.99967 | -4.51135 |
| DLGAP1 | 1.702293 | 2.950909 | 4.355952 | 0.007897 | 0.99967 | -4.51207 |
| FBXO40 | -1.819280 | 3.047120 | -4.331629 | 0.008076 | 0.99967 | -4.51232 |
| MTTP | 1.858959 | 2.622126 | 4.322305 | 0.008145 | 0.99967 | -4.51241 |
| HSD17B6 | -2.162608 | 2.712990 | -4.286391 | 0.008420 | 0.99967 | -4.51278 |
| DAPK2 | 1.596199 | 3.579492 | 4.275641 | 0.008504 | 0.99967 | -4.51290 |
| CXCL2 | -1.666197 | 8.427512 | -4.262629 | 0.008608 | 0.99967 | -4.51303 |
| PNMT | 1.783249 | 4.408842 | 4.233458 | 0.008845 | 0.99967 | -4.51334 |
| NKX2-5 | -1.715748 | 4.158686 | -4.205410 | 0.009080 | 0.99967 | -4.51364 |
| IL26 | 1.874847 | 2.262745 | 4.171978 | 0.009370 | 0.99967 | -4.51401 |
| GAS8-AS1 | 1.692954 | 3.510579 | 4.162492 | 0.009454 | 0.99967 | -4.51412 |
| VIP | 2.177127 | 2.212347 | 4.149443 | 0.009571 | 0.99967 | -4.51426 |
| RETN | -2.372506 | 2.798015 | -4.129145 | 0.009757 | 0.99967 | -4.51449 |
| CYP3A7 | -1.569571 | 3.701910 | -4.112984 | 0.009908 | 0.99967 | -4.51467 |
| IL4 | 2.160002 | 1.853710 | 4.107360 | 0.009961 | 0.99967 | -4.51474 |
| SPIB | -2.000422 | 2.585868 | -4.102892 | 0.010003 | 0.99967 | -4.51479 |
| ZNF141 | 2.135246 | 2.809634 | 4.097414 | 0.010056 | 0.99967 | -4.51485 |
| IL15RA | -1.521658 | 4.198729 | -4.078259 | 0.010241 | 0.99967 | -4.51507 |
| H3C10 | -1.464482 | 4.543378 | -4.066768 | 0.010354 | 0.99967 | -4.51520 |
| ARNTL | -1.660667 | 4.861006 | -4.044016 | 0.010583 | 0.99967 | -4.51547 |
| SIM1 | 1.475476 | 2.575443 | 3.997711 | 0.011066 | 0.99967 | -4.51602 |
| SEMA4A | -2.050037 | 4.458967 | -3.985250 | 0.011200 | 0.99967 | -4.51617 |
| FAT2 | 1.716559 | 3.814461 | 3.981266 | 0.011244 | 0.99967 | -4.51622 |
| MYCN | -1.466063 | 2.631856 | -3.911011 | 0.012041 | 0.99967 | -4.51709 |
| GAMT | -1.492193 | 2.320596 | -3.901484 | 0.012154 | 0.99967 | -4.51721 |
| COL6A3 | -1.564752 | 3.277032 | -3.892403 | 0.012263 | 0.99967 | -4.51733 |
| HOXC8 | 1.811045 | 3.459496 | 3.884250 | 0.012362 | 0.99967 | -4.51743 |
| VNN1 | -1.725419 | 1.832558 | -3.880520 | 0.012408 | 0.99967 | -4.51748 |
| CD79B | -1.382810 | 3.747445 | -3.877571 | 0.012444 | 0.99967 | -4.51752 |
| CCL17 | -1.672584 | 3.661320 | -3.856726 | 0.012703 | 0.99967 | -4.51779 |
| OXT | 1.378040 | 3.958868 | 3.854315 | 0.012733 | 0.99967 | -4.51782 |
| FBXO22 | 1.630242 | 2.988838 | 3.828751 | 0.013060 | 0.99967 | -4.51815 |
| DNASE2B | 1.639427 | 3.471362 | 3.827248 | 0.013079 | 0.99967 | -4.51817 |
| FKBPL | -1.524865 | 4.448141 | -3.823668 | 0.013126 | 0.99967 | -4.51822 |
| PNLIPRP1 | 1.360366 | 3.612113 | 3.822435 | 0.013142 | 0.99967 | -4.51823 |
| CCR8 | -1.544676 | 3.614579 | -3.820398 | 0.013168 | 0.99967 | -4.51826 |
| RDH8 | -1.640005 | 3.401614 | -3.817464 | 0.013207 | 0.99967 | -4.51830 |
| ATF3 | -1.459135 | 7.048859 | -3.770100 | 0.013846 | 0.99967 | -4.51893 |
| CALCRL | 1.380435 | 3.804904 | 3.769870 | 0.013850 | 0.99967 | -4.51894 |
| DIAPH3 | -1.700115 | 2.409575 | -3.755479 | 0.014051 | 0.99967 | -4.51913 |
| FABP2 | 1.619777 | 3.797847 | 3.738918 | 0.014287 | 0.99967 | -4.51936 |
| SCN11A | -1.664054 | 3.030911 | -3.714359 | 0.014645 | 0.99967 | -4.51970 |
| PPARA | 1.687456 | 3.287497 | 3.695674 | 0.014925 | 0.99967 | -4.51996 |
| GCNT1 | -1.815021 | 3.638645 | -3.668666 | 0.015340 | 0.99967 | -4.52035 |
| REM1 | -1.517722 | 2.991136 | -3.653254 | 0.015583 | 0.99967 | -4.52057 |
| ITGA2B | -1.613591 | 3.502016 | -3.635410 | 0.015869 | 0.99967 | -4.52083 |
| OCA2 | 1.614360 | 3.483767 | 3.622507 | 0.016080 | 0.99967 | -4.52102 |
| DCLK1 | 1.491377 | 5.552586 | 3.616599 | 0.016178 | 0.99967 | -4.52110 |
| NBR2 | -1.761998 | 3.461229 | -3.615446 | 0.016197 | 0.99967 | -4.52112 |
| OLR1 | -1.300671 | 3.762579 | -3.601822 | 0.016426 | 0.99967 | -4.52132 |
| NR3C2 | -1.530090 | 2.656355 | -3.601624 | 0.016429 | 0.99967 | -4.52133 |
| RBPJL | -1.497991 | 3.721971 | -3.595736 | 0.016529 | 0.99967 | -4.52141 |
| CHRNB4 | 1.305948 | 3.166996 | 3.594564 | 0.016549 | 0.99967 | -4.52143 |
| LY75 | 1.731261 | 1.708581 | 3.560490 | 0.017141 | 0.99967 | -4.52194 |
| NSG1 | -1.744022 | 2.801902 | -3.521479 | 0.017849 | 0.99967 | -4.52254 |
| SULT1C2 | -1.367910 | 5.535396 | -3.502500 | 0.018206 | 0.99967 | -4.52283 |
| IL6R | 2.090551 | 3.857051 | 3.500831 | 0.018237 | 0.99967 | -4.52286 |
| OR2J2 | 1.838313 | 2.475536 | 3.496401 | 0.018322 | 0.99967 | -4.52293 |
| ESR1 | -1.302823 | 3.854367 | -3.460056 | 0.019033 | 0.99967 | -4.52350 |
| IL2 | 1.556023 | 2.550007 | 3.452429 | 0.019186 | 0.99967 | -4.52363 |
| CCL22 | -1.281423 | 3.174159 | -3.451365 | 0.019208 | 0.99967 | -4.52364 |
| LINC01558 | 1.392855 | 3.964769 | 3.445226 | 0.019332 | 0.99967 | -4.52374 |
| C1QTNF3 | 1.337096 | 2.797657 | 3.440150 | 0.019436 | 0.99967 | -4.52382 |
| CD180 | 1.863954 | 4.190508 | 3.435505 | 0.019531 | 0.99967 | -4.52390 |
| EPPIN | -1.771277 | 3.103619 | -3.432508 | 0.019593 | 0.99967 | -4.52395 |
| ZMAT3 | 1.485393 | 5.516643 | 3.432080 | 0.019602 | 0.99967 | -4.52395 |
| NOL4 | 1.561421 | 2.928102 | 3.429225 | 0.019661 | 0.99967 | -4.52400 |
| LRP2 | -1.591192 | 3.103559 | -3.415320 | 0.019952 | 0.99967 | -4.52423 |
| NFE2 | -1.484679 | 2.620857 | -3.414174 | 0.019976 | 0.99967 | -4.52425 |
| FRY | 1.598540 | 2.840110 | 3.389352 | 0.020508 | 0.99967 | -4.52466 |
| CASP4LP | 1.635144 | 2.130581 | 3.372400 | 0.020881 | 0.99967 | -4.52494 |
| ERAP2 | -2.081548 | 2.531116 | -3.365009 | 0.021046 | 0.99967 | -4.52506 |
| CITED2 | -1.414779 | 5.125343 | -3.345014 | 0.021500 | 0.99967 | -4.52540 |
| ENPEP | 1.519549 | 1.741553 | 3.342978 | 0.021547 | 0.99967 | -4.52543 |
| LILRA2 | 1.850068 | 2.994810 | 3.333692 | 0.021762 | 0.99967 | -4.52559 |
| AANAT | -1.287315 | 1.573543 | -3.320513 | 0.022072 | 0.99967 | -4.52582 |
| RAPGEF4 | -1.538371 | 2.124807 | -3.319176 | 0.022104 | 0.99967 | -4.52584 |
| GALNT3 | 1.658567 | 2.084385 | 3.318294 | 0.022124 | 0.99967 | -4.52586 |
| GPR173 | -2.544731 | 3.735475 | -3.299828 | 0.022568 | 0.99967 | -4.52618 |
| CRABP1 | 1.336080 | 3.204125 | 3.298394 | 0.022603 | 0.99967 | -4.52620 |
| DCC | 1.719955 | 4.162514 | 3.288064 | 0.022856 | 0.99967 | -4.52638 |
| PSG2 | 1.734059 | 4.097736 | 3.271121 | 0.023277 | 0.99967 | -4.52668 |
| CLEC11A | -1.748712 | 3.900405 | -3.269213 | 0.023325 | 0.99967 | -4.52671 |
| GABRA6 | 1.383677 | 1.525262 | 3.258266 | 0.023603 | 0.99967 | -4.52691 |
| CSF1R | -1.491076 | 3.929933 | -3.249790 | 0.023821 | 0.99967 | -4.52706 |
| AKAP12 | 1.159115 | 7.598715 | 3.242893 | 0.024000 | 0.99967 | -4.52718 |
| CAMK1D | -1.317396 | 3.425283 | -3.229403 | 0.024354 | 0.99967 | -4.52742 |
| PAGE1 | 1.779364 | 3.350010 | 3.218728 | 0.024639 | 0.99967 | -4.52762 |
| ANKRD2 | -1.321801 | 2.974553 | -3.209539 | 0.024887 | 0.99967 | -4.52778 |
| EPHX2 | -1.369019 | 2.895365 | -3.197204 | 0.025224 | 0.99967 | -4.52801 |
| AIRE | 1.136783 | 3.365664 | 3.156256 | 0.026381 | 0.99967 | -4.52877 |
| KCND3 | 1.162170 | 2.867195 | 3.143964 | 0.026741 | 0.99967 | -4.52900 |
| PLA2G3 | 1.374875 | 3.476666 | 3.143405 | 0.026757 | 0.99967 | -4.52901 |
| PIK3CG | 2.018967 | 1.491613 | 3.126274 | 0.027267 | 0.99967 | -4.52934 |
| CDK5R2 | 1.862674 | 2.117597 | 3.112808 | 0.027676 | 0.99967 | -4.52959 |
| TPH1 | -1.980218 | 1.910894 | -3.101460 | 0.028026 | 0.99967 | -4.52981 |
| SOX11 | -1.538018 | 2.400982 | -3.094983 | 0.028228 | 0.99967 | -4.52994 |
| PPBPP2 | -1.277878 | 2.200106 | -3.087866 | 0.028451 | 0.99967 | -4.53008 |
| IL36G | -1.470128 | 3.465908 | -3.085527 | 0.028525 | 0.99967 | -4.53012 |
| NGB | -1.146688 | 1.781034 | -3.081176 | 0.028663 | 0.99967 | -4.53021 |
| HSD17B2 | -1.609498 | 2.981970 | -3.074094 | 0.028890 | 0.99967 | -4.53035 |
| ATXN7 | 1.146005 | 4.909523 | 3.073386 | 0.028913 | 0.99967 | -4.53036 |
| WNT8B | 1.220850 | 2.271138 | 3.072236 | 0.028950 | 0.99967 | -4.53038 |
| ROR2 | 1.142728 | 3.529148 | 3.066438 | 0.029137 | 0.99967 | -4.53050 |
| IRAK3 | 1.318045 | 2.555250 | 3.059717 | 0.029356 | 0.99967 | -4.53063 |
| AMFR | 1.185653 | 5.784661 | 3.055178 | 0.029505 | 0.99967 | -4.53072 |
| H2AC11 | -1.505935 | 2.027947 | -3.052386 | 0.029597 | 0.99967 | -4.53077 |
| BCL2L11 | -2.193485 | 1.940989 | -3.047483 | 0.029759 | 0.99967 | -4.53087 |
| DLG4 | -1.344020 | 3.538486 | -3.043213 | 0.029902 | 0.99967 | -4.53096 |
| AGRP | 1.255813 | 4.743168 | 3.040435 | 0.029995 | 0.99967 | -4.53101 |
| MED16 | -1.307874 | 4.400943 | -3.035592 | 0.030158 | 0.99967 | -4.53111 |
| IL33 | -1.628982 | 2.699184 | -3.029543 | 0.030362 | 0.99967 | -4.53123 |
| TREM2 | 1.331200 | 2.734958 | 3.013024 | 0.030930 | 0.99967 | -4.53156 |
| MCHR1 | 1.389503 | 3.444093 | 3.001262 | 0.031341 | 0.99967 | -4.53180 |
| SLC18A3 | -1.342243 | 4.201318 | -2.999473 | 0.031404 | 0.99967 | -4.53184 |
| EVI2A | 1.144430 | 3.647534 | 2.996533 | 0.031508 | 0.99967 | -4.53190 |
| FLT4 | 1.244858 | 3.457938 | 2.986967 | 0.031849 | 0.99967 | -4.53209 |
| PTGFR | -1.718604 | 1.452940 | -2.985625 | 0.031897 | 0.99967 | -4.53212 |
| REG1A | -1.812640 | 3.357608 | -2.964462 | 0.032668 | 0.99967 | -4.53256 |
| MFNG | 1.071609 | 4.893027 | 2.964429 | 0.032669 | 0.99967 | -4.53256 |
| MOG | -2.312898 | 2.389655 | -2.960597 | 0.032811 | 0.99967 | -4.53264 |
| MT1G | -1.352730 | 3.219059 | -2.947673 | 0.033294 | 0.99967 | -4.53291 |
| CD6 | 1.560338 | 4.529594 | 2.947478 | 0.033301 | 0.99967 | -4.53291 |
| CCT8L2 | 1.304822 | 2.730512 | 2.942792 | 0.033478 | 0.99967 | -4.53301 |
| MST1L | 1.445041 | 2.413768 | 2.941338 | 0.033534 | 0.99967 | -4.53304 |
| XPNPEP2 | 1.187757 | 4.452735 | 2.941250 | 0.033537 | 0.99967 | -4.53304 |
| EGR1 | -3.152159 | 6.169903 | -2.932882 | 0.033857 | 0.99967 | -4.53322 |
| MAFK | 1.389694 | 3.603176 | 2.932549 | 0.033869 | 0.99967 | -4.53323 |
| RECQL5 | -1.186428 | 3.996129 | -2.925343 | 0.034148 | 0.99967 | -4.53338 |
| SHMT1 | -2.186171 | 4.902100 | -2.924451 | 0.034182 | 0.99967 | -4.53340 |
| HK2 | -1.295654 | 2.901365 | -2.918956 | 0.034396 | 0.99967 | -4.53351 |
| CXCL11 | -1.095774 | 2.721953 | -2.901676 | 0.035080 | 0.99967 | -4.53388 |
| TFPI2 | 1.236062 | 7.762684 | 2.890914 | 0.035513 | 0.99967 | -4.53411 |
| MTAP | -1.805333 | 2.456929 | -2.889131 | 0.035585 | 0.99967 | -4.53415 |
| KIR2DL4 | 1.194480 | 4.616029 | 2.888235 | 0.035621 | 0.99967 | -4.53417 |
| CYBB | -1.178443 | 3.655060 | -2.882855 | 0.035841 | 0.99967 | -4.53429 |
| FTCD | 1.561591 | 4.444806 | 2.881460 | 0.035898 | 0.99967 | -4.53432 |
| TPPP3 | -1.300787 | 2.428498 | -2.879842 | 0.035965 | 0.99967 | -4.53435 |
| IFNA14 | 1.806620 | 4.090482 | 2.873960 | 0.036207 | 0.99967 | -4.53448 |
| SLC10A2 | 1.167601 | 3.201543 | 2.870679 | 0.036344 | 0.99967 | -4.53455 |
| JUNB | -1.040818 | 6.241939 | -2.869938 | 0.036374 | 0.99967 | -4.53457 |
| SORBS1 | 1.377318 | 1.710994 | 2.869047 | 0.036411 | 0.99967 | -4.53459 |
| CAMK2A | 1.098504 | 4.012139 | 2.866979 | 0.036498 | 0.99967 | -4.53463 |
| GPR65 | 1.039701 | 2.930909 | 2.858395 | 0.036858 | 0.99967 | -4.53482 |
| DLX2 | -1.622733 | 2.259740 | -2.855939 | 0.036962 | 0.99967 | -4.53488 |
| SLC4A10 | 1.212199 | 3.602955 | 2.855782 | 0.036969 | 0.99967 | -4.53488 |
| FPR1 | 1.633549 | 3.761618 | 2.850098 | 0.037211 | 0.99967 | -4.53501 |
| CLIC2 | -1.227951 | 2.054832 | -2.834895 | 0.037866 | 0.99967 | -4.53534 |
| ITGB3 | -1.003180 | 4.640355 | -2.832355 | 0.037977 | 0.99967 | -4.53540 |
| ST3GAL1 | -1.372371 | 2.291033 | -2.829651 | 0.038096 | 0.99967 | -4.53546 |
| IL1RAP | 1.908761 | 3.289353 | 2.817764 | 0.038621 | 0.99967 | -4.53572 |
| FGF17 | 1.568494 | 3.828620 | 2.816914 | 0.038659 | 0.99967 | -4.53574 |
| APOA4 | 1.062873 | 3.067575 | 2.793732 | 0.039707 | 0.99967 | -4.53627 |
| PBX1 | -1.303014 | 6.307429 | -2.789309 | 0.039911 | 0.99967 | -4.53637 |
| NR4A2 | -1.126170 | 5.473511 | -2.774263 | 0.040613 | 0.99967 | -4.53671 |
| IL13RA2 | 1.609665 | 2.584705 | 2.766752 | 0.040968 | 0.99967 | -4.53688 |
| B3GALNT1 | 0.979011 | 1.046547 | 2.766504 | 0.040980 | 0.99967 | -4.53689 |
| LRRC2 | 1.296836 | 3.263811 | 2.760217 | 0.041280 | 0.99967 | -4.53703 |
| TAAR3P | 1.267664 | 2.596313 | 2.750782 | 0.041735 | 0.99967 | -4.53725 |
| POU3F2 | 1.406017 | 2.930458 | 2.745762 | 0.041980 | 0.99967 | -4.53737 |
| MYH11 | -1.807253 | 2.417911 | -2.743385 | 0.042096 | 0.99967 | -4.53742 |
| RNF19A | -1.989003 | 4.428676 | -2.741856 | 0.042171 | 0.99967 | -4.53746 |
| FOSL2 | -2.829590 | 4.288164 | -2.723829 | 0.043066 | 0.99967 | -4.53788 |
| PDGFB | 1.125430 | 4.179040 | 2.723000 | 0.043108 | 0.99967 | -4.53790 |
| ENTPD5 | -1.160465 | 4.851342 | -2.699122 | 0.044328 | 0.99967 | -4.53847 |
| PPP2R2B | -1.218708 | 3.999790 | -2.687157 | 0.044954 | 0.99967 | -4.53875 |
| IL6 | -1.126744 | 6.066946 | -2.677702 | 0.045456 | 0.99967 | -4.53898 |
| RHAG | -1.126314 | 3.443005 | -2.673915 | 0.045658 | 0.99967 | -4.53907 |
| TSBP1 | -1.138243 | 2.284799 | -2.672905 | 0.045712 | 0.99967 | -4.53910 |
| KLF2 | -1.628316 | 6.155825 | -2.660541 | 0.046382 | 0.99967 | -4.53940 |
| SEMA6B | -1.443289 | 4.116110 | -2.660462 | 0.046386 | 0.99967 | -4.53940 |
| SIGLEC7 | -1.253883 | 3.447485 | -2.659190 | 0.046455 | 0.99967 | -4.53943 |
| TACSTD2 | 1.564787 | 2.278596 | 2.643302 | 0.047333 | 0.99967 | -4.53982 |
| CCND2 | 1.336361 | 3.404786 | 2.635562 | 0.047767 | 0.99967 | -4.54001 |
| H3C1 | -1.344086 | 2.386597 | -2.630870 | 0.048033 | 0.99967 | -4.54012 |
| GABPA | -1.125160 | 3.424878 | -2.622527 | 0.048509 | 0.99967 | -4.54033 |
| GZMB | 1.581471 | 3.390680 | 2.621922 | 0.048543 | 0.99967 | -4.54034 |
| NEU2 | -1.032405 | 1.701922 | -2.620298 | 0.048637 | 0.99967 | -4.54038 |
| HRH4 | 1.214223 | 3.268373 | 2.618487 | 0.048741 | 0.99967 | -4.54043 |
| ZP2 | -0.924844 | 1.833899 | -2.610738 | 0.049190 | 0.99967 | -4.54062 |
| ITGB7 | 1.374227 | 2.292595 | 2.606785 | 0.049421 | 0.99967 | -4.54072 |
| SMUG1 | -1.016763 | 4.593943 | -2.605438 | 0.049500 | 0.99967 | -4.54076 |
| PYCARD | 1.096448 | 2.935785 | 2.603784 | 0.049597 | 0.99967 | -4.54080 |
| ZNRD2 | -1.005372 | 5.645650 | -2.601464 | 0.049733 | 0.99967 | -4.54085 |
| TFF2 | 0.980359 | 3.989036 | 2.600378 | 0.049797 | 0.99967 | -4.54088 |
| GAD2 | 1.362519 | 4.491439 | 2.599290 | 0.049862 | 0.99967 | -4.54091 |
| RPS20 | 1.049939 | 3.624004 | 2.598513 | 0.049907 | 0.99967 | -4.54093 |

**Supplemental Figures**

**
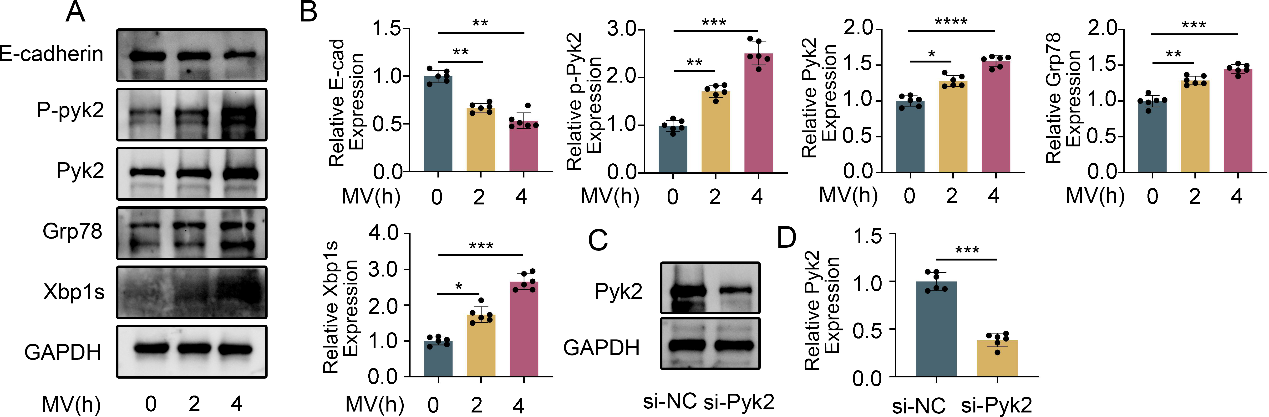
**

**Figure S1. Expression level of related proteins in lung tissue**

(A and B) Western blot results showed the expression of E-cadherin, Grp78, Xbp1s, p-Pyk2, and Pyk2 in mouse lung tissue in different groups.

(C and D) Western blot results showed knockdown efficiency of Pyk2 in mouse lung tissue.

Data are mean (SD), n = 6; *p < 0.05, **p < 0.01, ***p < 0.005, ****p < 0.001.

**
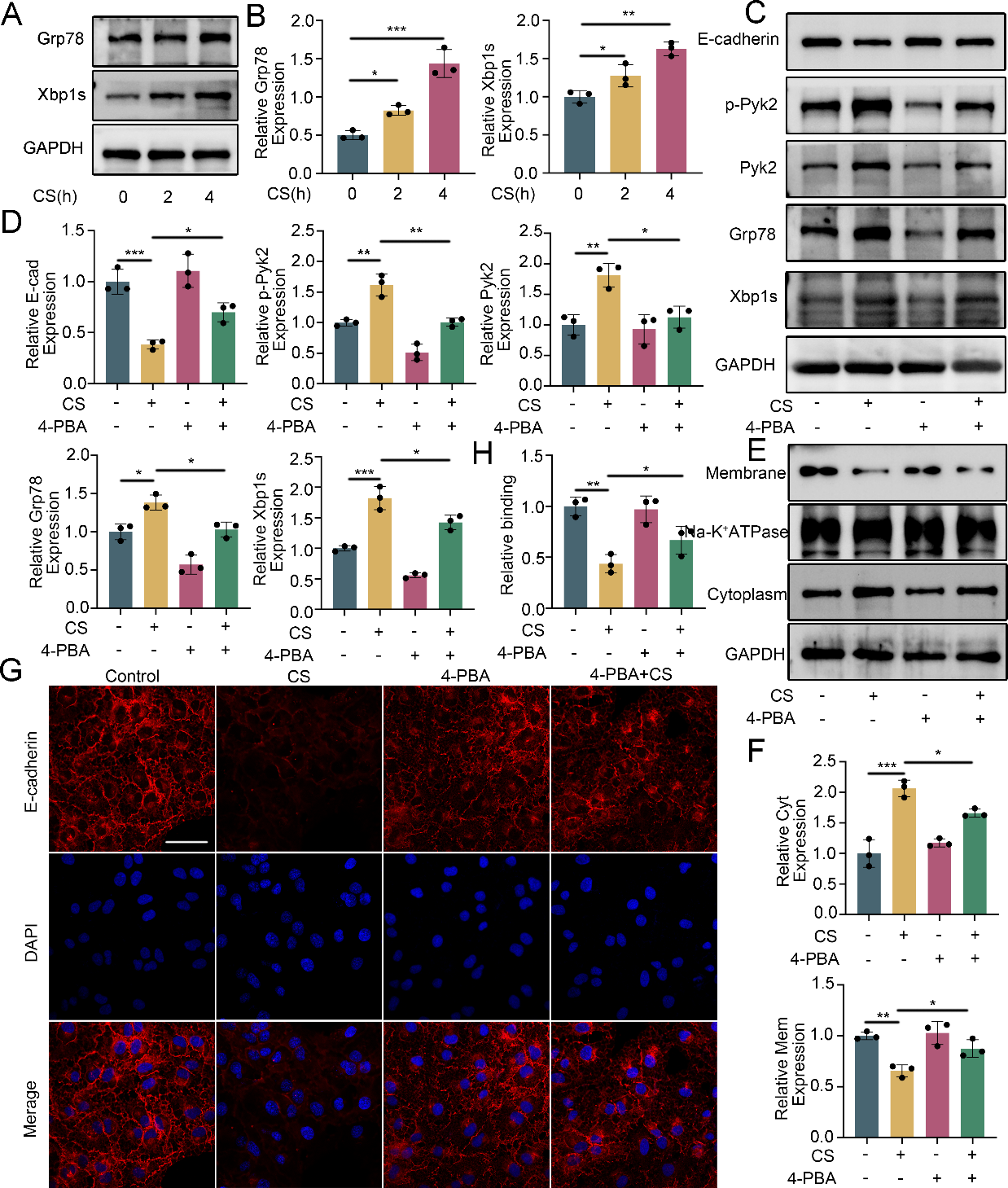
Figure S2. Cyclic stretching induces ER stress resulting in changes of Pyk2 and E-cadherin**

(A and B) Western blot results showed the expression of Grp78 and Xbp1s in different groups. (C and D) 4-PBA is an ER stress inhibitor. Western blot results show the expression of E-cadherin in different groups. (E and F) Western blot results showed the expression of E-cadherin in the cell membrane and cytoplasm in different groups.

(G and H) Immunofluorescence results showed the expression of E-cadherin in different groups, Blue: DAPI. Red: E-cadherin, scale bar: 30 μm.

Data are mean (SD), n=3; *p < 0.05, **p < 0.01, ***p < 0.005.
